# Combining next-generation sequencing and single-molecule sequencing to explore brown plant hopper responses to contrasting genotypes of japonica rice

**DOI:** 10.1186/s12864-019-6049-7

**Published:** 2019-08-29

**Authors:** Jing Zhang, Wei Guan, Chaomei Huang, Yinxia Hu, Yu Chen, Jianping Guo, Cong Zhou, Rongzhi Chen, Bo Du, Lili Zhu, Danax Huanhan, Guangcun He

**Affiliations:** State Key Laboratory of Hybrid Rice, College of Life Sciences, Wuhan University, Wuhan, China

**Keywords:** Brown plant hopper, Host resistance, RNA-seq, Single-molecule, real-time sequencing, Transcriptome, FoxO signaling pathway

## Abstract

**Background:**

The brown plant hopper (BPH), *Nilaparvata lugens*, is one of the major pest of rice (*Oryza sativa*). Plant defenses against insect herbivores have been extensively studied, but our understanding of insect responses to host plants’ resistance mechanisms is still limited. The purpose of this study is to characterize transcripts of BPH and reveal the responses of BPH insects to resistant rice at transcription level by using the advanced molecular techniques, the next-generation sequencing (NGS) and the single-molecule, real-time (SMRT) sequencing.

**Results:**

The current study obtained 24,891 collapsed isoforms of full-length transcripts, and 20,662 were mapped to known annotated genes, including 17,175 novel transcripts. The current study also identified 915 fusion genes, 1794 novel genes, 2435 long non-coding RNAs (lncRNAs), and 20,356 alternative splicing events. Moreover, analysis of differentially expressed genes (DEGs) revealed that genes involved in metabolic and cell proliferation processes were significantly enriched in up-regulated and down-regulated sets, respectively, in BPH fed on resistant rice relative to BPH fed on susceptible wild type rice. Furthermore, the FoxO signaling pathway was involved and genes related to BPH starvation response (*Nlbmm*), apoptosis and autophagy (*caspase 8*, *ATG13*, *BNIP3* and *IAP*), active oxygen elimination (*catalase*, *MSR*, *ferritin*) and detoxification (*GST*, *CarE*) were up-regulated in BPH responses to resistant rice.

**Conclusions:**

The current study provides the first demonstrations of the full diversity and complexity of the BPH transcriptome, and indicates that BPH responses to rice resistance, might be related to starvation stress responses, nutrient transformation, oxidative decomposition, and detoxification. The current result findings will facilitate further exploration of molecular mechanisms of interaction between BPH insects and host rice.

**Electronic supplementary material:**

The online version of this article (10.1186/s12864-019-6049-7) contains supplementary material, which is available to authorized users.

## Background

During more than 350 million years of co-evolution, herbivorous insects have developed various mechanisms to feed on plants, and counter diverse direct and indirect defenses against their feeding that plants have evolved [1–3]. Piercing and sucking insects comprise a large group of herbivores using plant phloem as their target and the transported fluid as their primary nutrient source. While feeding, these insects also reportedly secrete saliva into the plant tissue, which is thought to contain enzymes or effectors that interfere with plant defense responses [4]. Resistance and defense response of plants to piercing and sucking insects, which have been extensively studied, include systems that recognize effectors and trigger a stronger resistance reaction against herbivores. Sealing sieve tube, strengthening cell wall, and producing secondary metabolites and defense-related proteins are major resistance mechanisms against phloem-sucking insects. Changes in membrane potentials, Ca^2+^-signaling, levels and distributions of reactive oxygen species, kinase activities and phytohormones are known to play essential roles in early plant immune response to insect [5–8]. However, our understanding of insect responses to resistant host plants, particularly at genomic and transcriptomic levels, is still limited.

The brown plant hopper (BPH), *Nilaparvata lugens*, is a piercing and sucking insect pest of rice (*Oryza sativa*) which belongs to family Delphacidae, order Hemiptera, class Insecta of phylum Arthropoda. BPH feeding causes damages directly to rice plants mainly during crop reproductive stage. When rice crop is seriously harmed by BPH, the phenomenon of “hopper burn” occurs, resulting in significant reduction in rice yield, or even complete losses. BPH also indirectly injures rice plants by transmitting viruses [9, 10]. As well as being a major crop, rice is a model plant for genome research [11]. Hence, multiple omics approaches, such as functional genomic, transcriptomic, proteomic, and metabonomic analyses have been used in attempts to elucidate rice defenses to BPH [12–14]. Several BPH-resistance genes in rice have been isolated via the map-based cloning [15–18]. For example, *Bph6* (which confers broad resistance to BPH and white-backed plant hopper) has been shown to encode a previously uncharacterized protein that interacts with the exocyst subunit OsEXO70E1, thereby increasing exocytosis, and induces coordinated activation of cytokinin, salicylic acid, and jasmonic acid signaling pathways in response to BPH feeding [16].

With the rapid development of sequencing technology, research on insect omics is increasing [19, 20]. High-throughput, highly scalable, next-generation sequencing now allows entire genomes or transcriptomes to be sequenced simultaneously, at feasible cost [21]. Moreover, single-molecule, real-time (SMRT) sequencing can provide full-length transcript information and accurate isoform annotation [22, 23], thereby enabling comprehensive analysis of complex transcriptomes [24–27]. Thus, as reported here, the current study have combined next-generation and SMRT sequencing to characterize transcripts of BPH (including tissues of antenna, stylet and salivary gland, which play crucial roles in interactions with host plants [28, 29]) and reveal the responses of BPH insects to resistant rice by comparing insects fed on resistant *Bph6*-transgenic and susceptible wild type rice plants.

## Results

### Sequencing and characterizing full-length isoforms of BPH

**Fig. 1:**
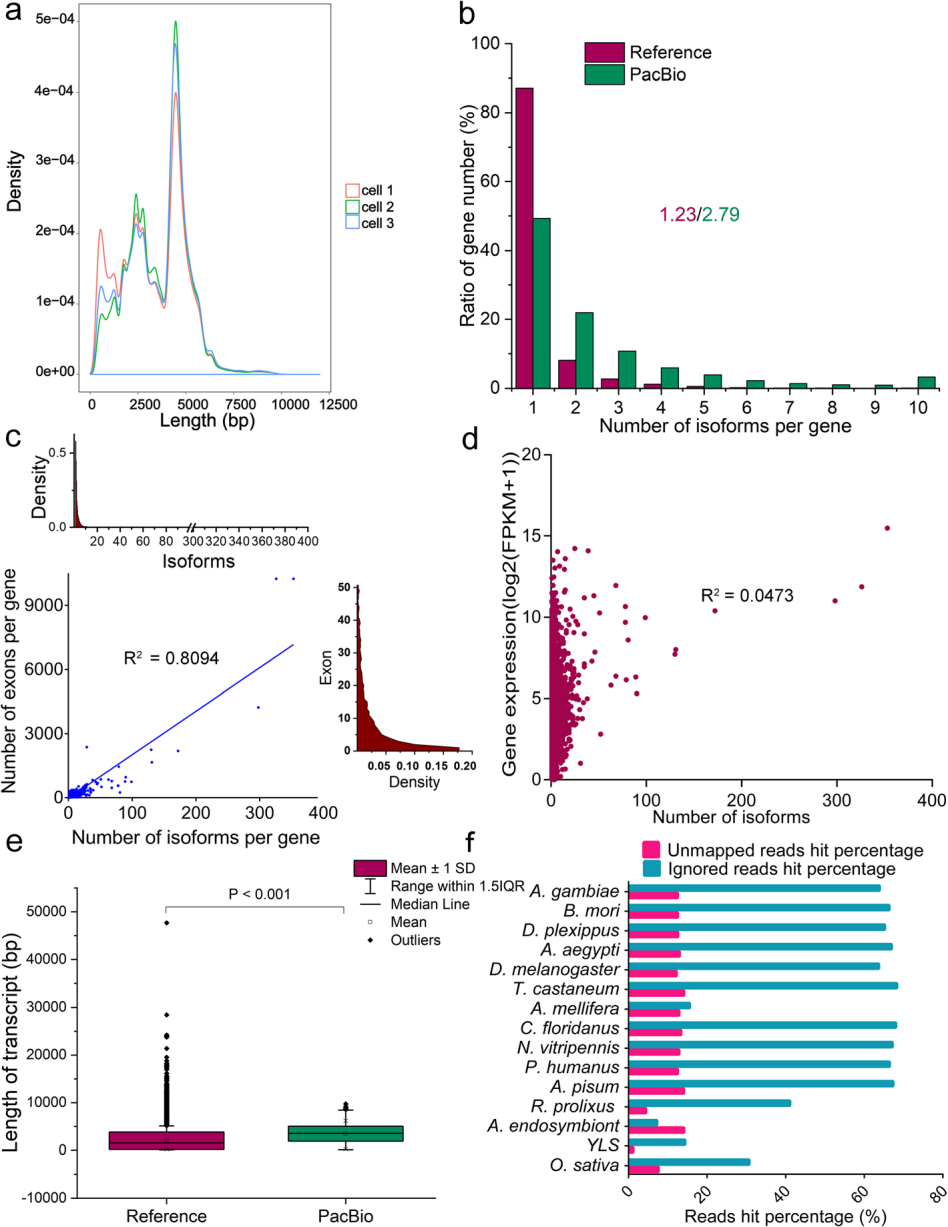
Characteristics of transcript isoforms in *N. lugens* detected by PacBio sequencing. **a**, Distribution of insert reads’ length in three SMRT cells. **b**, Comparison of number of isoforms per gene between transcripts in the reference genome NilLug1.0 and PacBio sequencing dataset. **c**, Correlation between the number of isoforms per gene and number of exons per gene in the PacBio sequencing data (lower left graph), density profile of the number of exons per gene (right graph) and density distribution of the number of isoforms per gene (upper left graph). **d**, Correlation between the number of isoforms per gene and gene expression level according to PacBio sequencing results. **e**, Comparison of length of transcripts in the reference genome NilLug1.0 and PacBio sequencing isoforms (with significance of difference determined by Wilcoxon rank sum test). **f**, Percentages of unmapped and filtered PacBio reads mapped to the genomes of 15 species using Blastx with an E < 1e-5

SMRTbell libraries from mixed tissues of 80 female adults of BPH biotype 1 were sequenced using a PacBio Sequel system with a V2 reagent kit and three SMRT cells, yielding 15.69 Gb clean data. The insert length distribution in each cell was very similar (Fig. 1a, Table 1). The current study obtained 436,057 reads of inserts (ROI), with 11.31 passes on average. In total, 376,347 (86.31%) reads were classified as full-length (FL) based on the presence of barcoded primers and poly A tails (Table 1). After Iterative Clustering for Error Correction (ICE) [30] followed by Long Read DBG Error Correction (Lordec) [31] using the current study RNA sequencing (RNA-Seq) data (see below) (Additional file 1: Figure S1), 176,807 sequences totaling 574,066,263 bp were obtained. These sequences were mapped to the BPH reference genome NilLug1.0 (https://www.ncbi.nlm.nih.gov/genome/?term=txid108931 [Organism:noexp]) using the Genomic Mapping and Alignment Program (GMAP) [32]. 172,329 (98.94%) sequences were aligned to the BPH reference genome, and 2631 were identified as reads of 915 fusion genes. The current study obtained 24,891 collapsed isoforms of full-length transcripts after redundancy removal, of which 20,662 were mapped to 7395 annotated genes in the reference genome. Of the 20,662 mapped transcripts, 3487 (16.88%) were identical to the original transcripts, and most (17,175, 83.12%) were novel transcripts of known genes in BPH genome.

**Table 1:** Data yield of single-molecule, real-time sequencing

Sample	Cell 1	Cell 2	Cell 3	All
Number of reads of insert (ROI)	135,059	168,798	132,200	436,057
Mean length of ROI (bp)	3258	3569	3533	3453
Mean number of passes	12.25	10.61	11.06	11.31
Number of five prime reads	126,260	158,466	122,763	407,489
Number of three prime reads	126,962	157,275	123,642	407,879
Number of poly-A reads	126,683	156,800	123,158	406,641
Number of full-length reads	117,577	145,568	113,202	376,347
Number of full-length non-chimeric reads	114,878	142,212	109,810	366,900
Mean full-length non-chimeric read length (bp)	3088	3432	3367	3295

According to the current study PacBio data, 3749 of the 7395 annotated genes (50.70%) encoded more than one isoform, compared to 2545 (12.85% in 19,806 protein-coding genes) in the reference genome NilLug1.0. The average number of isoforms per gene (2.79) in the PacBio dataset was more than twice the number (1.23) in the reference genome (Fig. 1b). In addition, 237 of the 7395 annotated genes (3.20%) had more than 10 isoforms, and three had extremely high numbers of isoforms: PB.6723 (LOC111054078, 353 isoforms), PB.450 (LOC111049488, 326 isoforms), and PB.5698 (LOC111051863, 298 isoforms). Furthermore, the number of isoforms per gene was positively correlated with the number of exons per gene (*R*^*2*^ = 0.8094), but not expression level (*R*^*2*^ = 0.0473) (Fig. 1c, d). This is consistent with reported patterns in maize (*Zea mays*) [24]. By comparing the sequence structure of full-length transcripts with reference genome annotations, we identified 41,331 extension (5′ or 3′ end) in 5815 corresponding genes (Additional file 2: Table S1). The length of transcripts in the current study PacBio data (median, 3598 bp) is longer on average than that (median, 1561 bp) in the reference genome NilLug1.0 (Fig. 1e). The results clearly indicated that the full-length transcripts obtained by SMRT improved the annotation quality of the reference genome of *N. lugens*.

The unmapped reads (1847; 1.06% of the total) and filtered PacBio reads (29,073; 16.87%) were used as query sequences and further mapped to the genomes of 12 BPH-related species (*Bombyx mori*, *Danaus plexippus*, *Anopheles gambiae*, *Aedes aegypti*, *Drosophila melanogaster*, *Tribolium castaneum*, *Apis mellifera*, *Camponotus floridanus*, *Nasonia vitripennis*, *Pediculus humanus*, *Rhodnius prolixus*, and *Acyrthosiphon pisum*), two facultative microbial endosymbionts (*yeast-like symbiont* and *Arsenophonus endosymbiont*) [33] and the host rice *O. sativa* using Blastx with an E < 1e-5 cutoff (Additional file 3, Table S2). In addition to a few sequences with matches in host rice and endosymbiotic bacteria genomes, ‘hits’ were obtained for a number of the sequences in genomes of the BPH-related species (Fig. 1f). The results suggested a strong likelihood that these sequences are genuinely encoded in the BPH genome.

### Alternative splicing detection and verification

**Fig. 2:**
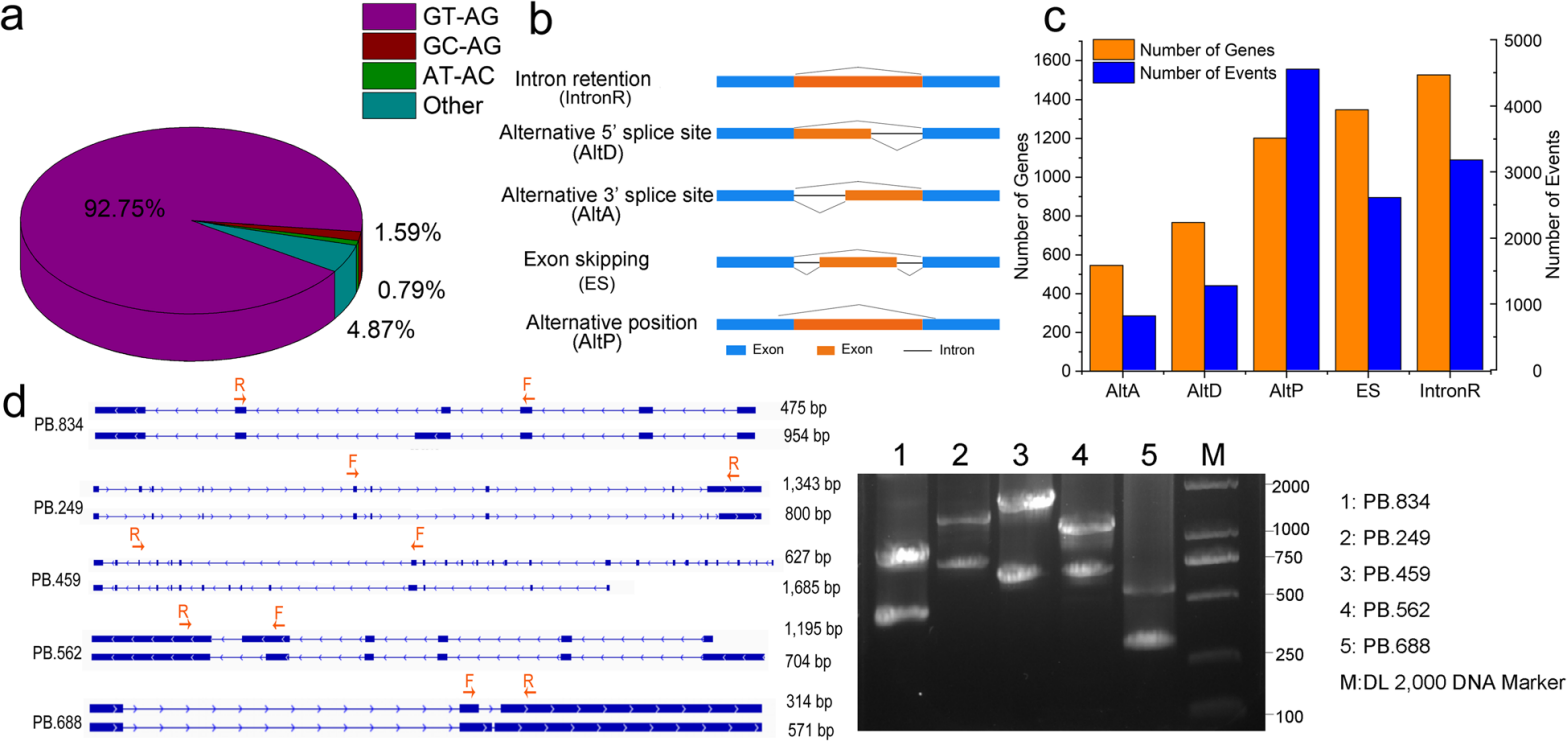
Alternative splicing detection and verification in transcripts of *N. lugens*. **a**, Distribution of intron boundary types. **b**, Models for five types of alternative splicing. **c**, Classification and statistics of alternative splicing events. **d**, RT-PCR validation of AS events for five genes. The full-length transcript structure of each isoform is shown on the left. Blue boxes and blue lines represent exons and introns, respectively. Orientations from left to right and vice versa indicate that the transcript is located in positive and negative chains, respectively. PCR primers (F, forward and R, reverse) are displayed on the first isoform of each gene. The length of PCR products is shown to the right of the structure of each transcript. Agarose gel electrophoresis bands in the right figure show the PCR result and DNA marker

In higher eukaryotic organisms, most genes are interrupted by introns. It is necessary to remove introns from the pre-messenger RNA accurately to form the mature messenger RNA. The intron boundary sequences at the 5′ and 3′ ends are recognized and processed by spliceosome component. Using a Perl script to analyze PacBio isoforms and reference transcripts, we detected 349 intron boundary sequence types in BPH genes. However, the highly conserved GT-AG type [34], accounted for 92.75% of the total number of detected boundaries (Fig. 2a; Additional file 4: Table S3).

The multiplicity of splicing sites enables the generation of multiple isoforms of transcripts of the same gene through alternative splicing, thereby greatly extending the diversity of transcripts and proteins in eukaryotic organisms, and their regulatory complexity [35–37]. For example, previous analyses based on RNA-seq data have shown that transcripts of 31 and 25% of all genes in *Drosophila melanogaster* and *Caenorhabditis elegans*, respectively, are subject to alternative splicing [38, 39]. The current study PacBio full-length isoform data provide the first overall view of alternative splicing events in BPH. The current study detected 20,356 alternative splicing events from 3594 genes, accounting for 18.15% of all protein-coding genes (19,806) in BPH genome (Additional file 5: Table S4), and alternative splicing of 3119 genes’ products was responsible for 5988 novel transcripts. These findings indicate that alternative splicing plays an important role in transcript diversity in BPH. Using a custom Python script [40], five types of alternative splicing were identified: exon skipping (ES), alternative 3′ splice site (AltA), alternative 5′ splice site (AltD), intron retention (IntronR), and alternative position (AltP) (Fig. 2b). The current study found that genes with IntronR events accounted for the largest proportion, followed by genes with ES events. AltP was most common frequent occurrence type of alternative splicing in BPH, followed by IntronR (Fig. 2c). By combined analysis of the proportion of genes affected and the number of occurrences in each AS type, the current study found that IntronR seems to be the major type of alternative splicing in BPH*,* which is consistent with reported patterns in *Drosophila melanogaster, Caenorhabditis elegans and Bombyx mori* [38, 39, 41].

The current study randomly selected five genes and verified the accuracy of detected alternative splicing events by reverse transcription polymerase chain reaction (RT-PCR) analysis and Sanger sequencing. The current study first analyzed sequences of full-length transcripts of these genes that would result from the alternative splicing events, then designed primers for RT-PCR amplification (Additional file 6: Table S5), and finally examined the products by agarose gel electrophoresis and Sanger sequencing. Both size of RT-PCR products and Sanger sequencing results matched transcripts detected by the single-molecule, real-time sequencing (Fig. 2d).

### Long non-coding RNA identification

**Fig. 3:**
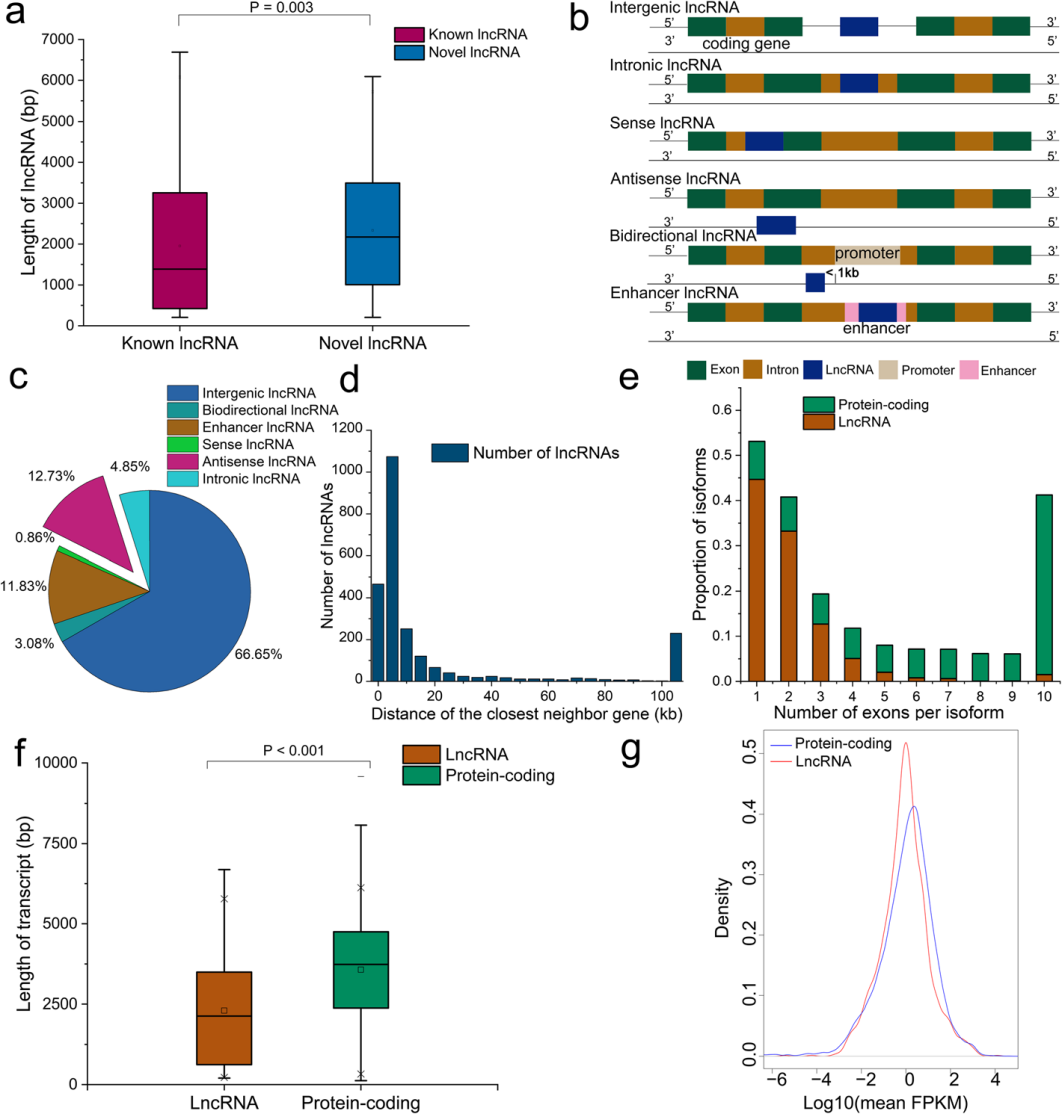
Characteristics of lncRNAs in *N. lugens*. **a**, Comparison of transcript length between the novel lncRNAs and known lncRNAs (with significance of difference determined by Wilcoxon rank sum test). **b**, Models for six types of lncRNA. **c**, Classification and statistics of lncRNA types. **d**, Frequency distribution of distances from the closest neighbor gene for lncRNA. **e** and **f**, Comparison of the number of exons per transcript and transcript lengths between lncRNAs and protein-coding transcripts, respectively (with significance of difference in latter determined by Wilcoxon rank sum test). **g**, Comparison of expression profiling between lncRNAs and protein-coding transcripts

Long non-coding RNAs (lncRNAs) are non-protein-coding transcripts that are longer than 200 nt and play important roles in gene expression regulation at various levels [42]. The BPH full-length isoforms that were aligned to the reference genome but lacked protein annotation were used to predict lncRNAs (2435 in total) (Additional file 7: Table S6). Of these predicted lncRNAs, 168 (median length, 1389 bp) matched annotated lncRNAs in the reference genome (which includes 1075 in total) (Additional file 7: Table S7), while 2267 (median length, 2172 bp) were novel (Fig. 3a; Additional file 7: Table S8). According to their positions in the genome, lncRNAs can be divided into six types: intergenic (lincRNA), intronic, sense, antisense, bidirectional and enhancer lncRNAs (Fig. 3b). The current study found that intergenic lncRNA (1623, 66.65%) was the most common type in BPH (Fig. 3c), in accordance with previous results of genome-wide lncRNA identification in BPH and *Bombyx mori* [43, 44]. Previously, 2470 lincRNAs were identified in *Apis cerana*, 1514 in *A. mellifera* and 1119 in *D. melanogaster* based on RNA-seq datasets [45, 46]*.* The results indicate that lincRNA is the most frequent type in insects. LincRNAs reportedly regulate the expression of neighboring protein-coding genes, probably through participation in a *cis-*regulatory network [47–49]. The current study found that 19.14% of *N. lugens* lncRNAs overlap with, and 54.42% are located within < 10 Kb of known protein-coding genes (Fig. 3d). The result indicate that *N. lugens* lncRNAs tend to be close to protein-coding genes, and thus may speculatively participate in their *cis*-regulation.

Analysis of these predicted lncRNAs’ structure showed that 77.91% (1897) had only one or two exons, almost five times as high as the corresponding proportion of protein-coding transcripts (16.00%, 3592). Moreover, only 0.70% of the lncRNAs (17) has more than 10 exons, far less than the corresponding proportion (39.68%, 8911) of protein-coding transcripts (Fig. 3e). The average lengths of the predicted lncRNAs and protein-coding transcripts are 2307 and 3565 bp, respectively (Fig. 3f). Expression profiling confirmed that lncRNAs are less highly expressed than protein-coding transcripts (Fig. 3g). Thus, the current study results clearly indicate that lncRNAs have fewer exons per transcript and lower expression levels than protein-coding transcripts in BPH, in accordance with previous analyses of transcription patterns in both BPH and mammals [43, 50].

### Detection of novel genes and fusion transcripts

**Fig. 4:**
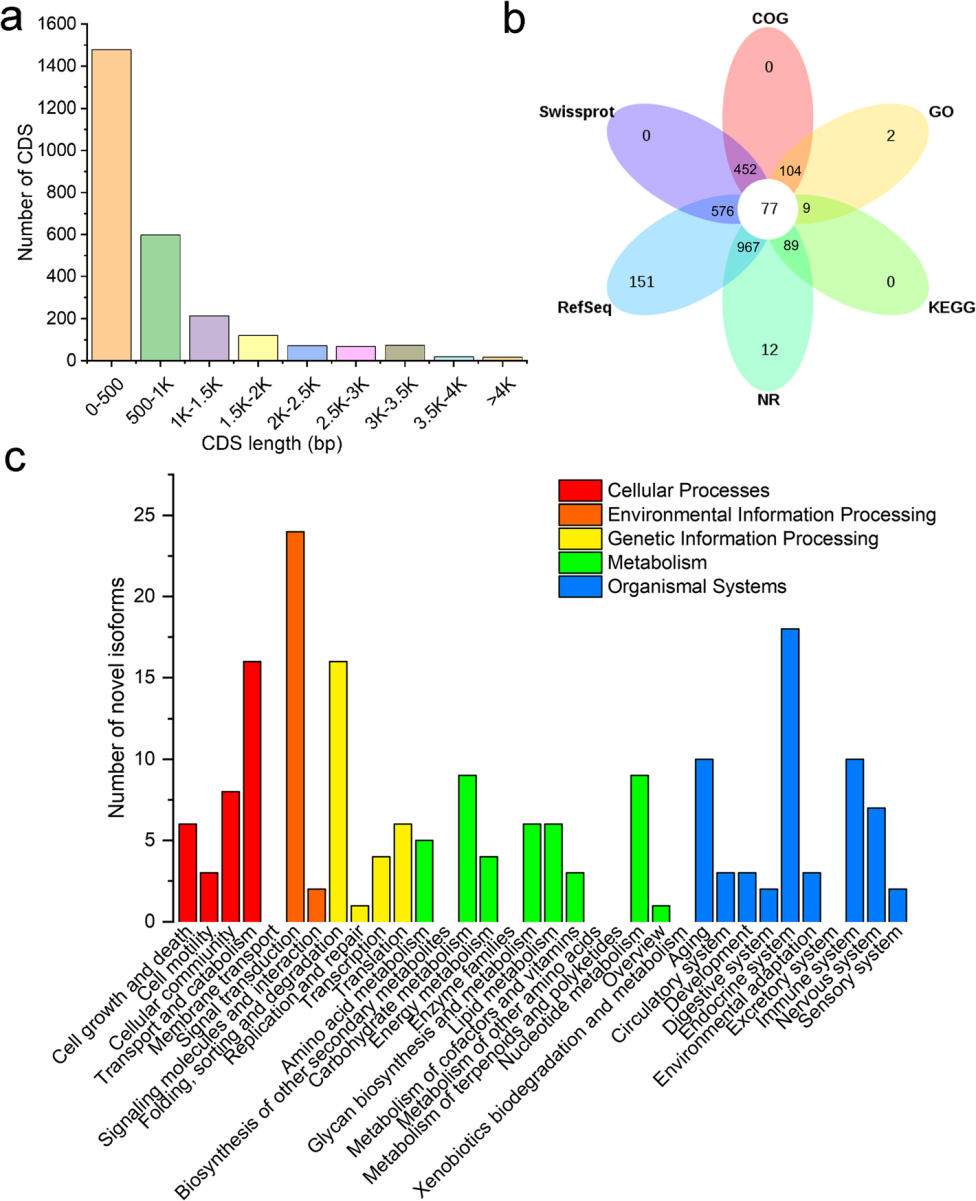
Novel genes detected in *N. lugens*. **a**, CDS length distribution of novel genes. **b**, Ven diagram showing function annotations of novel genes in six databases. **c**, KEGG pathway classification of novel genes

A novel gene refers here to a gene putatively encoding a detected transcript that does not match any annotated gene in the BPH reference genome. From analysis of the current study PacBio data, we predicted 2664 coding sequences (CDS) from 1794 novel genes (Additional file 8: Table S9 and Table S10), with an average length of 819 bp (Fig. 4a). Among these 1794 novel genes, homologues of 1213 (67.61%) were functionally annotated in at last one of the following databases: Non-redundant (Nr), Swiss-Prot, Kyoto Encyclopedia of Genes and Genomics (KEGG), Gene Ontology (GO), RefSeq and Clusters of Orthologous Group (COG). Moreover, homologues of 77 genes were annotated in all six databases (Fig. 4b; Additional file 9). KEGG classification analysis showed that functions of the novel genes were mainly related to transport and catabolism, signal transduction, folding, sorting and degradation, carbohydrate metabolism, and endocrine system (Fig. 4c).

**Fig. 5:**
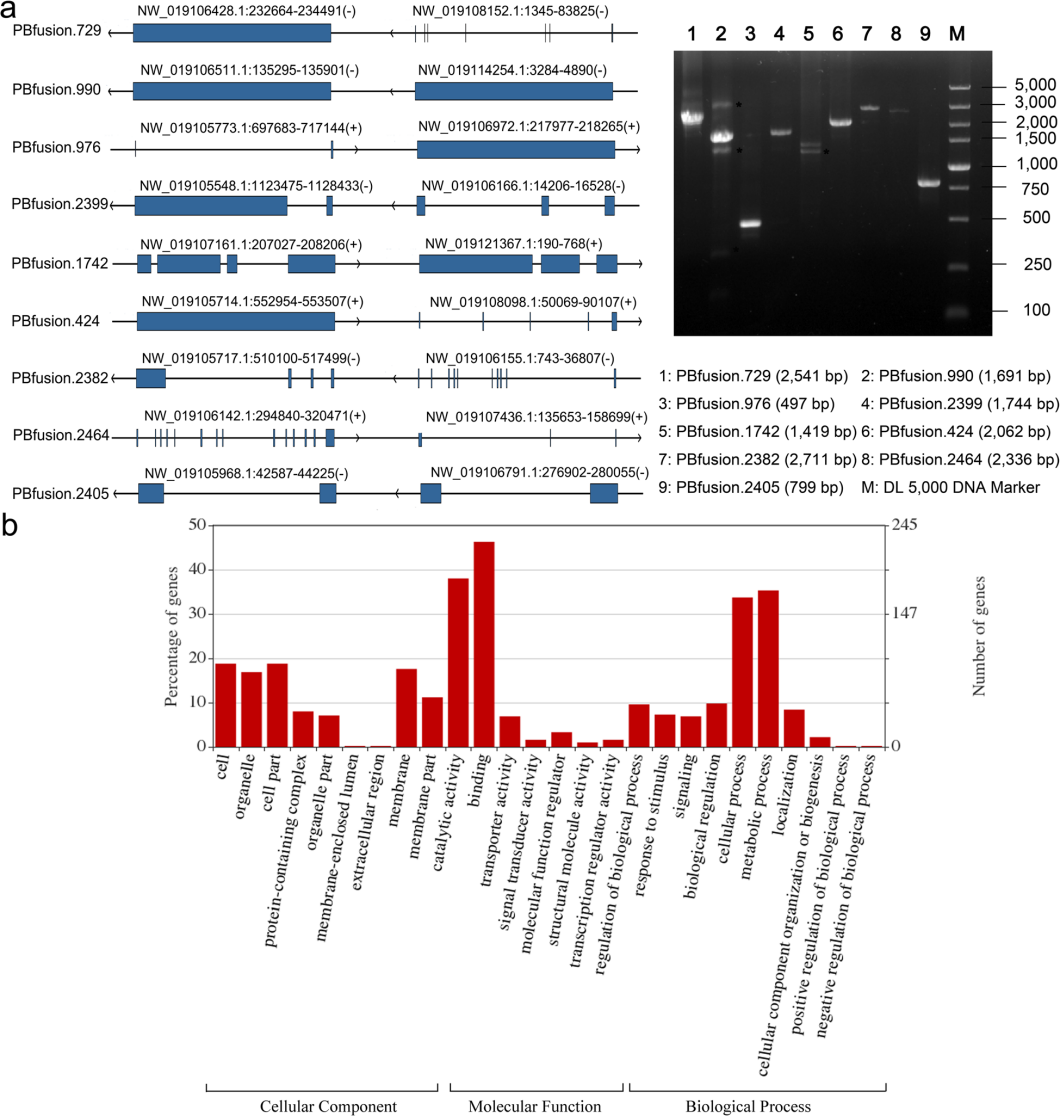
Verification and functional annotation of fusion genes in *N. lugens*. **a**, Verification of fusion genes by agarose gel electrophoresis, with structural sketches of the genes to the left (corresponding to loci indicated above the structures). Blue boxes and vertical lines represent exons, while introns are represented by black horizontal lines. Orientations from left to right and vice versa indicate that the transcript is located in positive and negative chains, respectively. Gel brands in the right figure show the PCR products and DNA marker. The length of each fusion transcript is shown after its ID. **b**, GO classification of fusion genes’ annotation

Fusion genes are formed by the cleavage and re-ligation of two different chromosomes, or cleavage and rearrangement of a chromosome, which binds normally separated transcriptional sequences [51]. Fusion genes are prevalent and common in cancer, and gene fusion is regarded as a distinct class of mutations [52], but there have been few analyses of gene fusion in insects. We detected 2631 reads supporting the presence of 915 fusion genes in the current study BPH samples (Additional file 10: Table S11). Of all the detected fusion genes, 417 (45.57%) fused sequences of annotated genes at different locations in the reference genome, 105 (11.48%) fused sequences of unannotated genes, and 393 (42.95%) fused sequences of annotated and unannotated genes (Additional file 10: Table S12). *Trans*-splicing links exons from two independent transcripts, generating chimeric RNA, which has been detected in most eukaryotes [53]. The fusion sites of different transcript are identical to their respective splice sites in the normal splicing process, which is the key indication of *trans*-splicing [24]. The current study found that fusion boundaries of 166 of the 417 transcripts representing fused sequences of annotated genes (27.82%) corresponded to junctions in normal splicing (Additional file 11). This suggests that parts of fusion transcripts resulting from fusion of sequences of annotated genes may be generated by the RNAs *trans*-splicing machinery, which augments the BPH transcriptome’s diversity and complexity. To further verify the fusion genes, nine fusion transcripts were randomly selected and validated by RT-PCR and Sanger sequencing analysis. Nine fusion transcripts were experimentally validated, which confirmed the authenticity of these transcripts (Fig. 5a; Additional file 6: Table S5). GO classification analysis of all fusion genes revealed that functions of most of them were related to binding, catalytic activity, metabolic processes, and cellular processes (Fig. 5b).

### Differentially expressed genes in plant hoppers fed on resistant and susceptible rice plants

The previous study has cloned the BPH-resistance gene *Bph6* in rice [16]. In this study, *Bph6* was expressed under the control of native promoter and transformed into BPH-susceptible Japonica rice variety Nipponbare by Agrobacterium tumefaciens-mediated transformation [54]. A T2 homozygous transgenic line (named *Bph6*-transgenic rice) with a single-copy insertion was used for BPH resistance evaluation (Additional file 12: Figure S2). The results indicated that *Bph6*-transgenic rice has high resistance to BPH, whereas wild-type Nipponbare plants are susceptible and died after BPH infestation. The current study then observed the performance of newly emerged female BPH adults that were allowed to feed, for 48 h, on both genotypes of rice plants, and found they had a significantly lower survival rate on *Bph6*-transgenic rice (62.83%) than on Nipponbare (95.11%) (Additional file 13: Figure S3a). Two simply measurable indicators of BPH feeding activity, weight gain and honeydew extraction [55], were also significantly lower during feeding on *Bph6*-transgenic rice (0.29 and 8.81 mg, respectively) than during feeding on Nipponbare (1.17 and 62.15 mg, respectively) (Additional file 13: Figure S3b, S3c). Moreover, the weight gain ratio of BPH insects fed on *Bph6*-transgenic rice (0.14) was only about one-fifth of that on Nipponbare (0.76) (Additional file 13: Figure S3d). Thus, feeding on the *Bph6*-transgenic rice plants induced significant resistance stress in BPH insects, and we used them and wild type Nipponbare plants to characterize gene regulation in responses to host plant resistance in BPH, via next generation RNA-sequencing.

**Fig. 6:**
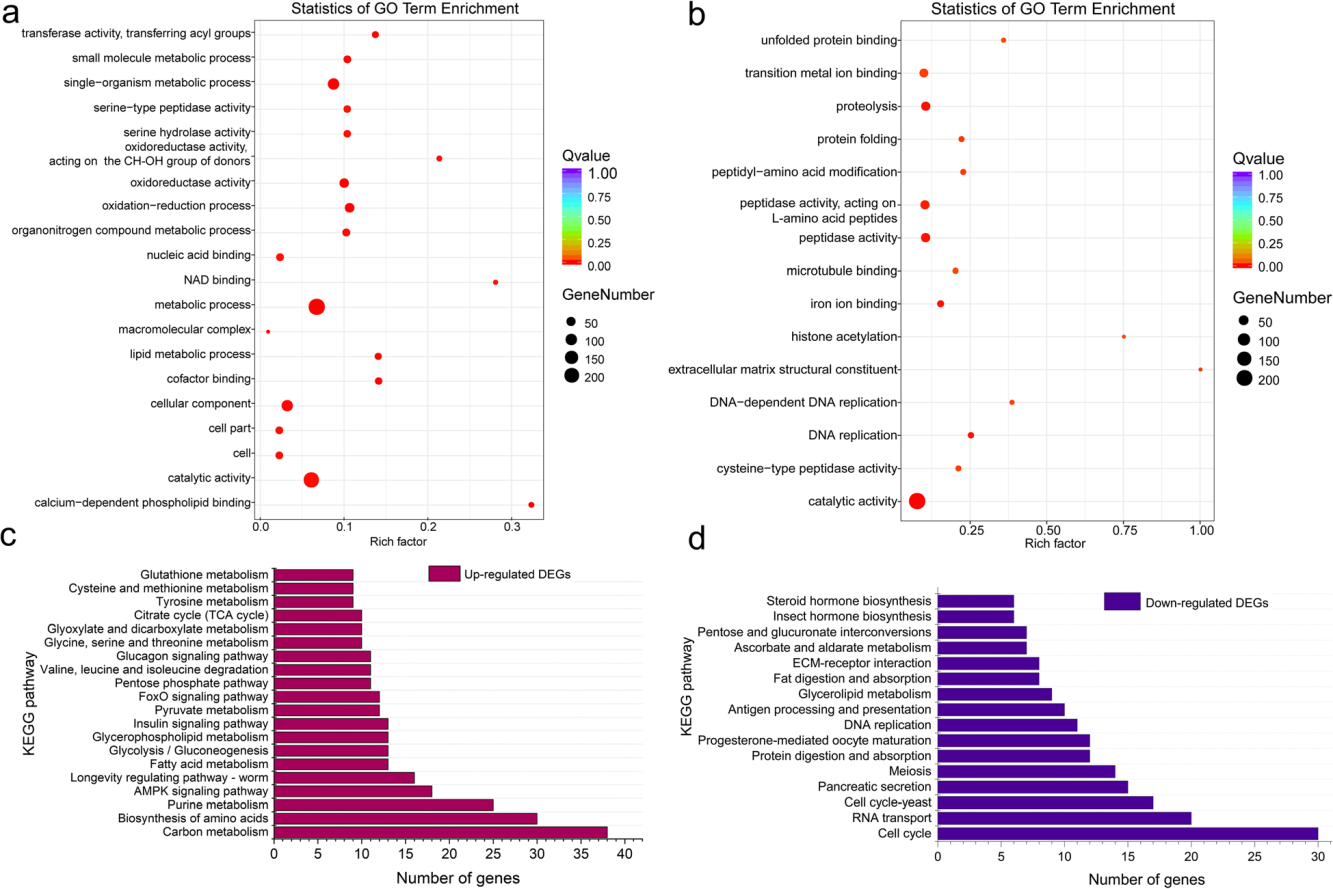
GO and KEGG enrichment analysis of DEGs. **a**, Twenty most highly enriched GO terms in up-regulated DEGs. **b**, All GO terms enriched in down-regulated DEGs. **c**, Twenty most highly enriched KEGG pathways in up-regulated DEGs. **d**, All KEGG pathways enriched in down-regulated DEGs

RNA samples from four biological replicates of insects fed on Nipponbare for 48 h (designated N-BPH) and others fed on *Bph6*-transgenic rice for 48 h (designated NB6-BPH) were sequenced using an Illumina HiSeq Xten PE150 platform (Table 2). Results of correlation analysis and Principal Component Analysis (PCA) showed that biological replicates correlated well (Additional file 14: Figure S4). Comparison of NB6-BPH and N-BPH gene expression profiles identified 1893 differentially expressed genes (DEGs) with fold change (FC) ≥ 2 and false discovery rate (FDR) < 0.01 (Additional file 15: Figure S5; Additional file 16: Figure S6). Among these genes, 895 were up-regulated, and 998 were down-regulated in insects fed on *Bph6*-transgenic rice relative to those fed on Nipponbare (Additional file 17: Tables S13 and S14). The DEGs were subsequently subjected to GO and KEGG enrichment analyses based on Q values (adjusted *P*-values) < 0.05 (Additional file 18: Tables S15 and S16; Additional file 19: Tables S17 and S18). They were classified into three main functional categories by GO enrichment analysis—molecular function (MF), cell composition (CC) and biological process (BP). As shown by the sets listed in Fig. 6a and b*,* the most strongly enriched GO terms in the up-regulated and down-regulated DEGs are associated with metabolic processes and catalytic activities, respectively.

**Table 2:** Statistics of RNA-seq data

Sample	Read length (bp)	Clean reads	Clean bases	Q30 Rate (%)
N-1	150	39,212,486	5,881,872,900	89.87
N-2	150	40,992,814	6,148,922,100	88.33
N-3	150	44,493,422	6,674,013,300	89.11
N-4	150	40,176,198	6,026,429,700	89.87
NB6–1	150	40,234,308	6,035,146,200	89.55
NB6–2	150	37,245,018	5,586,752,700	89.15
NB6–3	150	40,161,428	6,024,214,200	89.92
NB6–4	150	46,213,362	6,932,004,300	90.56

**Fig. 7:**
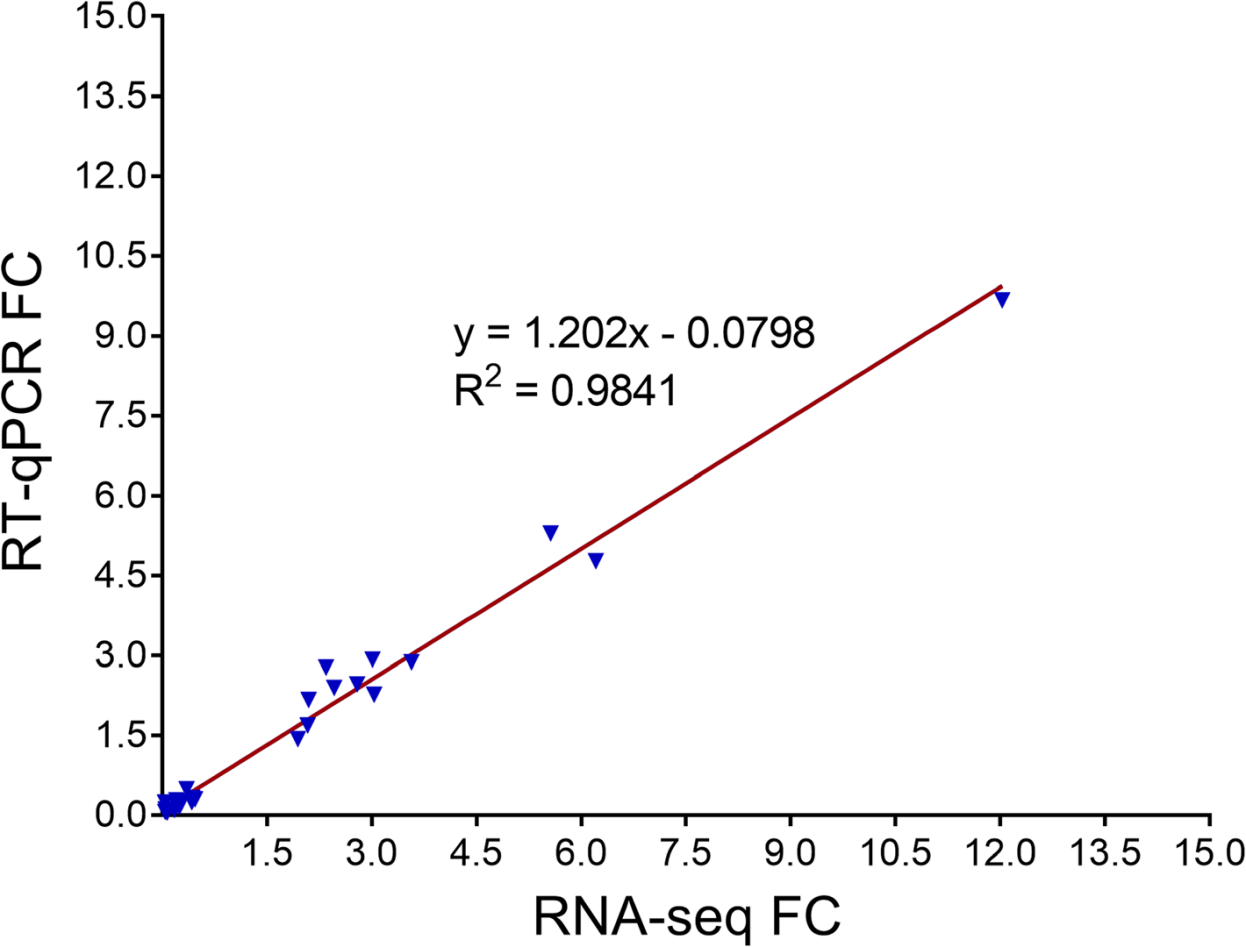
Concordance of expression fold changes of genes detected in RNA-seq and RT-qPCR analyses. The blue triangles represent 12 up-regulated genes and 15 down-regulated genes. BPH *actin* was used as an internal reference gene, three biological replicates were analyzed

KEGG pathway enrichment analysis (with a Q value threshold of < 0.05) showed that energy metabolism processes (including carbon metabolism, pentose phosphate pathway, glycolysis/gluconeogenesis, citrate cycle and fatty acid metabolism etc.) were the main up-regulated metabolic pathways. Moreover, up-regulated DEGs (but not down-regulated DEGs) were significantly enriched in the AMP-activated protein kinase (AMPK) signaling, FoxO signaling, longevity regulation and insulin signaling pathways (Fig. 6c). The down-regulated DEGs were mainly enriched in cell cycle and RNA transport. Moreover, genes involved in DNA replication, meiosis, and the digestion and absorption of fat and protein were also significantly enriched in down-regulated DEGs (Fig. 6d). These results clearly indicate that BPH mainly up-regulated metabolic processes and down-regulated cell proliferation at the transcriptional level in response to rice resistance stress. To verify the reliability of the DEGs, 12 up-regulated genes and 15 down-regulated genes were randomly selected for real-time quantitative PCR (RT-qPCR), and the results were in good agreement with the RNA-seq data (Fig. 7; Additional file 6: Table S5).

### The FoxO signaling pathway is involved in BPH responses to resistance stress

**Fig. 8:**
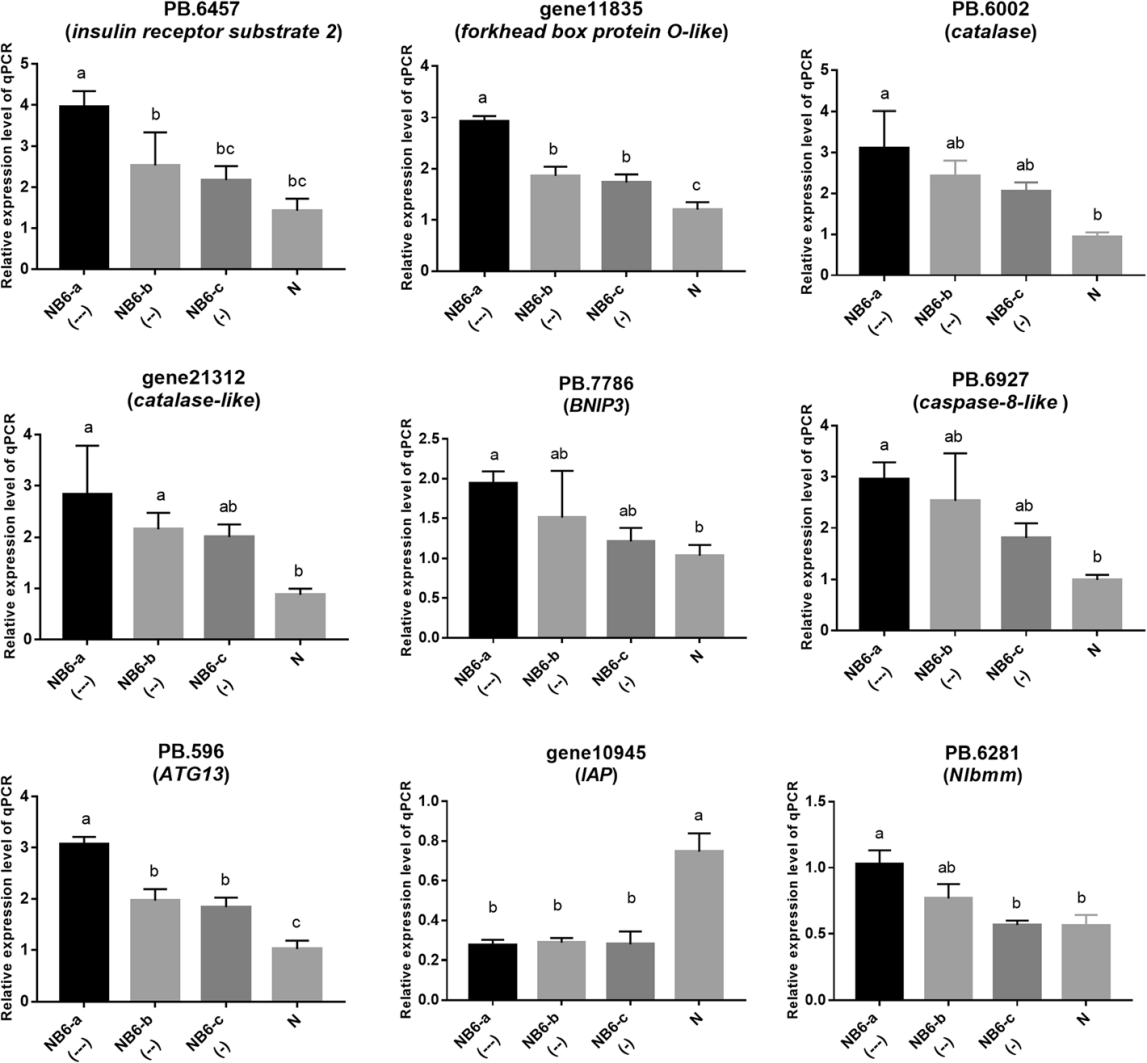
Expression of nine genes in BPH insects fed on resistant and susceptible rice plants. The top seven genes were involved in the FoxO signaling pathway. The last two genes represent caspase inhibitor (*IAP*) and lipase brummer (*Nlbmm*), respectively. N: BPH fed on Nipponbare susceptible rice for 48 h, with 2.0 mg weight gain. NB6-a(---), NB6-b(--) and NB6-c(-): BPH insects fed on *Bph6*-transgenic rice for 48 h with varying degrees of weight loss (− 0.4, − 0.2 and − 0.05 mg, respectively). Error bars represent SEM, *n* = 4 independent experiments, different letters above the bars indicate significant differences, according to one-way ANOVA test (*P* < 0.05)

As mentioned above, genes associated with the AMPK signaling, FoxO signaling, longevity regulation, and insulin signaling pathways were significantly enriched in the up-regulated DEGs in BPH insects fed on *Bph6*-transgenic rice. The FoxO signaling pathway is related to cell cycling, cell death, stress, detoxification, and other cellular processes [56]. FOXO transcription factors are regulated in response to nutrient deprivation by the AMPK pathway, and the AMPK-FOXO pathway plays a crucial role in the ability of a dietary restriction regimen to extend lifespan in *C. elegans* [57, 58]*.* In addition, a FOXO ortholog is a crucial mediator of the insulin signaling pathway in *Drosophila* [59]. Therefore, the current study verified the expression of seven up-regulated genes (*INRS*, *FOXO*, *caspase 8*, *ATG13*, *BNIP3*, *CAT* and *CAT-like*) involved in the FoxO signaling pathway in NB6-BPH insects by RT-qPCR (Fig. 8; Additional file 6, Table S5). The RT-qPCR results confirmed that the seven genes’ expression was up-regulated in all the BPH with inhibited weight gain on *Bph6*-transgenic rice (NB6-BPH), relative to N-BPH. It is well known that the FOXO transcription factor is activated and up-regulated under starvation conditions in insects [60, 61]. Starvation also induces increases in expression of *Nlbmm* (another up-regulated DEG in NB6-BPH: Fig. 8; Additional file 17: Table S13), while knockdown of *Nlbmm* confers significant hunger resistance in BPH [62]. These results all indicate that BPH responses to rice resistance might be related to starvation stress responses*.*

**Fig. 9:**
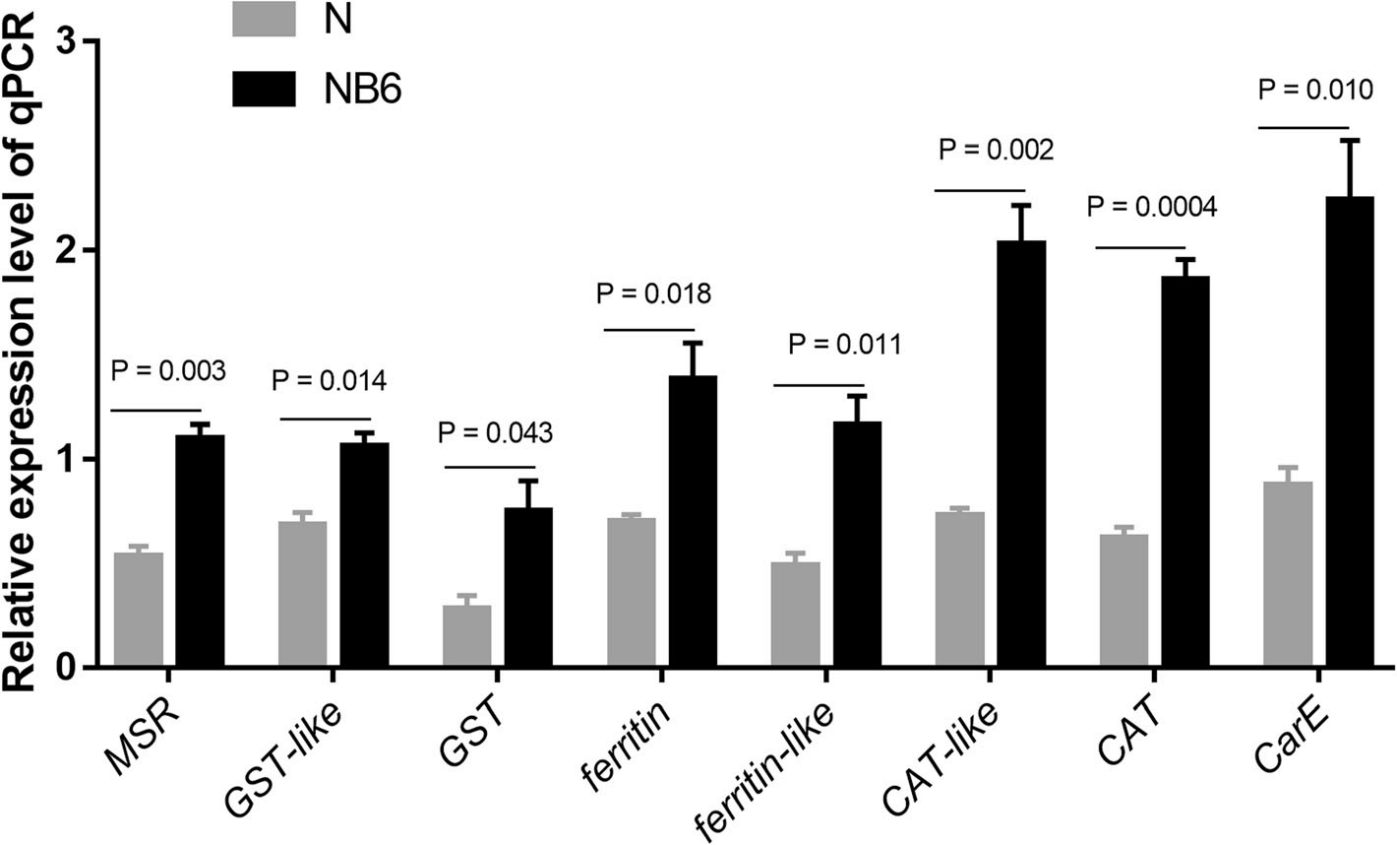
Differentially expressed genes related to active oxygen elimination and detoxification between N-BPH and NB6-BPH. N: BPH fed on Nipponbare susceptible rice for 48 h, with 2.0 mg weight gain. NB6: BPH fed on *Bph6*-transgenic rice for 48 h, with weight loss (− 0.5 mg). Error bars represent SEM, *n* = 3, calculating *P* Value with one-way ANOVA test

Caspase 8, ATG13 and BNIP3 are regulated by the FoxO signaling pathway and play important roles in apoptosis and autophagy [63–65]. These genes were significantly up-regulated, while *IAP* (which encodes an inhibitor of caspase [66, 67]), was significantly down-regulated in NB6-BPH (Fig. 8). These results suggest that feeding on resistant *Bph6*-transgenic plants may induce cell apoptosis and autophagy in BPH. Furthermore, we found that the expression of genes associated to active oxygen elimination and detoxification, such as *catalase* (*CAT*), *glutathione S-transferase* (*GST*), *methionine sulfoxide reductase* (*MSR*), *ferritin* and *carboxylesterases* (*CarE*) [68] were significantly up-regulated in NB6-BPH relative to N-BPH (Fig. 9; Additional file 6, Table S5). These results indicated that BPH’s adaptation to rice resistance stress may involve oxidative decomposition, and detoxification.

## Discussion

The current study combined next-generation and single-molecule sequencing to characterize the transcriptome of BPH biotype 1 and explore BPH responses to resistant rice plants. The results reveal the BPH transcriptome’s diversity and complexity, and show that analysis of full-length transcripts can improve annotation of the reference genome. The current study results also suggest that multiple pathways relating to starvation stress, active oxygen elimination and detoxification are regulated in BPH responses to resistant rice.

Nearly all of the single-molecule sequencing reads (98.94%) were mapped to the BPH reference genome NilLug1.0, indicating the high quality of the sequences. Among 20,662 isoforms annotated to 7395 genes, most (17,175; 83.12%) are novel transcripts that were not present in the reference genome database. The current study results illustrated the high diversity in the transcriptome of BPH that alternative splicing plays a crucial role in production of various BPH transcripts. The molecular diversity of insects’ innate immune system is known to involve alternative splicing [69], but the current study single-molecule sequencing of the BPH transcriptome showed that fusion transcripts also contribute to its diversity. In total, it detected 1794 novel transcripts without annotations. Thus, the current study findings extend knowledge of both transcript diversity in *N. lugens* and the mechanisms involved.

The current study results also extend knowledge of lncRNAs in BPH. In total, there are 1075 predicted lncRNAs in the BPH reference genome. However, the current study identified 2435 lncRNAs, of which 2267 were novel. In fruitfly and honeybee, lncRNA-regulated patterns include extensive *trans*-regulation by epiregulation of chromatin (re) organization or RNA sequestration in the nuclear compartment, and *cis*-regulation of neighboring protein-coding genes [70]. The current study found that intergenic lncRNA was the most common type in BPH. Whereas previous studies indicated that many intergenic lncRNAs are close to regulatory or developmental genes in the human, mouse and zebrafish genomes, and have potential *cis*-regulatory roles in protein-coding gene transcription [49]. The current study found that most of the BPH lncRNAs (1791; 73.56%) overlap with or are located within < 10 Kb of known protein-coding genes, suggesting that they likely participate in *cis*-regulation of these genes’ transcription. These research findings add to the current study comprehension of the complexity of transcriptional regulation in *N. lugens*, also indicate that both expansion of transcriptional subtypes from multiple exon genes, without increases in the number of genes, and regulation of protein-coding genes by lncRNAs, may be involved in BPH’s adaptations to the wide variation in rice [71]. The diversity and complexity of BPH transcriptome may also be required for the insects to adapt to various changes in environmental conditions during long-distance migrations, which are crucial elements of their life history and reproduction cycles [72].

The BPH is a monophagous insect of rice. In the process of long-term interaction between BPH and rice, rice has evolved resistance to BPH, which is controlled by the resistance genes. Previous studies have shown that during the long-term interactions between rice and its monophagous pest BPH, rice has evolved genes that confer varying degrees of resistance to BPH through sieve tube sealing, and production of both secondary metabolites and various kinds of proteins [6–8]. These genes include *Bph6*, which encodes a novel protein that locates on exocyst and participates in regulation of phytohormone signal pathways and excretion in rice cells. Deposition of callose in sieve tubes, strengthening of cell walls and production of phytoalexin have been observed in *Bph6* plants following infestation by BPH insects [16]. The current study verified that feeding on *Bph6*-transgenic rice plants reduced BPH insects’ survival rate and feeding activity, relative to feeding on susceptible wild type plants. The current study also discovered that BPH up-regulated metabolic processes and down-regulated cell proliferation at the transcriptional level when feeding on *Bph6*-transgenic rice plants. These energy metabolism process including carbon metabolism, pentose phosphate pathway, glycolysis /gluconeogenesis, citrate cycle and fatty acid metabolism were significantly enriched in up-regulated DEGs. Energy mobilization processes are generally strengthened in insects during starvation, embryogenesis, and immune responses that enhance their survival chances when food is scarce [73]. In contrast, endoreplication only occurs if there is adequate nutrition, at least in *D. melanogaster* cell cultures [74]. Thus, the down-regulation of DEGs involved in cell proliferation observed when BPH fed on resistant rice may relate to nutrient deficiency resulted from resistant stress.

In response to attacks by herbivorous insects that minimize damage, plants defense against insects involve activation of both chemical and physical feeding deterrents that lead to insects starvation [75, 76]. Presumably linked responses we detected in BPH responses to *Bph6*-transgenic rice plants included changes in expression of FoxO signaling pathway components. Overexpression of FOXO reportedly phenocopies starvation in *D. melanogaster*, suggesting that FOXO participates in adverse nutritional reactions [60, 61]. *Nlbmm* (known to be up-regulated in BPH starvation response [62]) was also up-regulated in NB6-BPH. These findings clearly indicate that some of the responses of BPH insects to short-term feeding on resistant rice plants are related to starvation stress. Other defenses of plants against insect herbivores include production of various secondary metabolites [77, 78]. However, one of the strategies for insects to overcome this problem is the detoxification of defense chemicals by oxidation, reduction, hydrolysis or conjugation of molecules [79, 80]. The current study found that genes related to active oxygen elimination and detoxification enzymes were up-regulated in BPH fed on *Bph6*-transgenic plants. Overall, the current study results imply that BPH responses to rice resistance stress include nutrient transformation, oxidative decomposition, and detoxification of xenobiotics.

## Conclusions

The current study concluded that the first indications of the full diversity and complexity of BPH transcriptome at the isoform level and showed that analysis of the full-length transcripts can improve annotation of the reference genome. In addition, analysis of DEGs between BPH insects fed on *Bph6*-transgenic plants and others fed on susceptible wild type plants indicated that the BPH responses to host resistance stress might relate to starvation stress, nutrient transformation, active oxygen elimination and detoxification. These findings may facilitate further elucidation of interactions between insect herbivores and host plants.

There were several limitations also noticed like, first, the current study revealed characteristics of alternative splicing, fusion genes and lncRNAs in BPH at the isoform level, but did not further explore their functions in interactions with rice. Second, the previous studies shown that FOXO, PI3K/Akt and MAPK pathways play significant roles in apoptotic responses of silkworm brain neurons to starvation [81], and starvation induced autophagic apoptosis in several insects [82–84]. Whereas, the current study found that genes related to starvation stress, apoptosis and autophagy were up-regulated in BPH fed on *Bph6*-transgenic plant, but the possibility that feeding on resistant rice induces autophagic cell apoptosis in BPH insects warrants further study in continuation to current research findings.

## Methods

### Brown plant hopper insects

BPH (*Nilaparvata lugens*) biotype 1 [85] insects were kept in the laboratory and maintained on 1-month-old plants of the susceptible rice cv Taichung Native1 (TN1) under controlled environmental conditions (26 ± 0.5 °C, 16 h light/8 h dark cycles) at the Genetics Institute, Wuhan University [55]. Newly emerged BPH female adults (about 16-17 days old) were used in experiments.

### Source of seeds and generation of transgenic rice plants

Susceptible wild type Taichung Native1 (TN1, IRRI Acc. No. 00105) and Nipponbare (IRGC Acc. No. 136196) rice seeds used in this study, and the seeds of *Bph6* carrying rice cultivar Swarnalata (IRRI Acc. No. 33964) were obtained from International Rice Research Institute.

The *Bph6*-transgenic line was maintained as previous protocol by Guo and coworkers [16]. The *Bph6* genome sequence was amplified from Swarnalata genomic DNA with B6G-F and B6G-R primers (Additional file 6: Table S5) and cloned into the vector pCAMBIA1300, then transformed into the susceptible recipient line Nipponbare rice through the *Agrobacterium*-mediated method. Southern blot analysis was used to determine the number of insertion copies in transgenic T_0_ lines obtained. Rice genomic DNA was digested with the restriction enzymes *DraI.* Hybridization of nucleic acids with a 728 bp biotinylated *hpt II* probe according to the operating manual of North2South Chemiluminescent Hybridization and Detection Kit (Thermo Scientific, Cat. No. 17097). Rice seedlings of the T_2_ homozygous progeny in the four-leaf stage from an independent *Bph6*-transgenic line were infected by second to third instar BPH nymphs for resistance evaluation, using the susceptible recipient line Nipponbare as a control. T_2_ homozygous progeny were identified by PCR analysis of their genomic DNA, using the *Bph6*-specific B6O-F and B6O-R primers (Additional file 6: Table S5), and the constructed vector and Nipponbare DNA as a positive and negative controls, respectively. A voucher specimen of the *Bph6*-transgenic line has been deposited in the China Center for Type Culture Collection (CCTCC No. P201907).

### Sample collection and RNA preparation

Susceptible wild type Nipponbare rice and *Bph6-*transgenic rice were used in BPH feeding experiments, as follows. Seeds of both types were sown in plastic cups (radius 4 cm, height 20 cm), five per cup, then cultivated in a greenhouse providing 30 ± 2 °C/14 h light and 28 ± 2 °C/10 h dark cycles at Wuhan University (30° north latitude and 114° eastern longitude). When rice plants of both types had grown from the seeds to the five-leaf stage (about 4 weeks after sowing) the performance of adult female BPH was measured [86]. The research evaluation involved data observation on BPH adult female with main traits viz. survival rate, weight gain, honeydew excretion, and weight gain ratio after feeding for 48 h on the plants.

In addition, 80 insects fed on Nipponbare for 48 h (N-BPH) [87] were randomly sampled in sets of four biological replicates, with 20 insects per replicate [88, 89]. Similarly, 80 insects fed on *Bph6-*transgenic rice for 48 h with the inhibited weight gain (NB6-BPH) were sampled for four biological replicates. As already mentioned the antenna, stylet, salivary gland and leg play important roles in BPH’s exploration and acquisition of food from rice plants [28, 29]. Therefore, the current study dissected heads and forelegs of BPH female insect, which contain these organs, from the sampled insects under a stereo microscope on ice. Tissues of the 20 insects in each group were mixed and total RNA was extracted from them, using an EASYspin tissue/cell RNA rapid extraction kit (Cat. #HF107-02). The Agilent 2100 Bioanalyzer (Agilent Technologies, USA) and agarose gel electrophoresis system (Lonza, Switzerland) were used to confirm the integrity of all RNA samples, and a NanoDrop 2000c Spectrophotometer (Thermo Scientific, USA) was used to measure RNA concentration.

### Illumina library construction and RNA sequencing

Sequencing libraries were constructed from high-quality RNA samples (OD260/280 = 1.8~2.2, OD260/230 ≥ 2.0, RIN ≥ 8, RNA > 1 μg) obtained from the replicates using a TruSeq® RNA Sample Preparation Kit v2 (Illumina, USA). PolyA mRNA was purified from total RNA (1.5 μg per sample), then fragmented. First-strand cDNA was synthesized using reverse transcriptase, random primers and these short fragments as templates, further the second-strand cDNA was synthesized. The resulting synthetic cDNA was end-repaired and adenylated, then Illumina Adaptors were ligated to prepare for hybridization. After that, the library fragments were purified and PCR amplification were performed following the standard library construction protocol [90]. At last, the library preparations were subjected to paired-end sequencing using an Illumina HiSeq X Ten platform.

### Library preparation and PacBio sequencing

Equal portions of the four total RNA samples obtained from NB6-BPH insects were pooled to construct a mixed RNA sample for PacBio sequencing. A SMARTer™ PCR cDNA Synthesis Kit (Takara, Cat. Nos. 634,925 and 634,926) was used for reverse transcription of the qualified RNA to cDNA, which was then subjected to PCR amplification using PrimeSTAR® GXL DNA polymerase (Takara, R050A) with optimized (12) cycles. Amplified products of suitable sizes (0.5-6 Kb fragments) were then amplified by large-scale PCR to obtain enough DNA, and a SMRTbell Template Prep Kit (Pacific Biosciences, 101-357-000) was used to construct a SMRTbell library. Sequel libraries with insertion fragments of 1-6 Kb were constructed and sequenced using a PacBio Sequel platform with V2.1 chemistry (Pacific Biosciences) and 10 h movies.

### Pipeline for PacBio data analysis

The quality of the sequencing reads was controlled by filtering out invalid reads and sequences with full pass less than 1 or accuracy less than 0.8, to obtain high quality insert reads. These reads were then divided into full-length, non-full-length and chimeric reads using the SMRT analysis software provided by Pacific Biosciences (http://www.pacb.com/products-and-services/analytical-software/smrt-analysis/). Subsequently, full-length non-chimeric (FLNC) reads were clustered and corrected by the Iterative Clustering for Error Correction (ICE) algorithm [30]. Lordec [31] was then applied to correct the clustering results with the RNA-seq data. All full-length reads were aligned with the BPH reference genome NilLug1.0 by GMAP [32] software, then collapsed by a Python script (collapse_isoforms_by_sam.py), with 0.9 minimum identity and 0.85 minimum coverage. MatchAnnot software (https://github.com/TomSkelly/MatchAnnot) was used to compare the alignment results with NilLug1.0 annotation.

### Identification of alternative splicing

Alternative splicing was identified and classified, using a Python script (https://github.com/Nextomics/pipeline-for-isoseq) [40], into five types: exon skipping (ES), alternative 3′ splice site (AltA), alternative 5′ splice site (AltD), intron retention (IntronR), and alternative position (AltP).

### Prediction of lncRNA and novel genes

An online tool (https://bitbucket.org/arrigonialberto/lncrnas-pipeline) was used to identify putative lncRNAs in the PacBio dataset that aligned with the reference genome but not annotated transcripts. The predicted lncRNAs were then compared with known lncRNAs in reference genome NilLug1.0. Those transcripts which could not be aligned to gene models in NilLug1.0 and not be predicted to be lncRNA were considered as novel transcripts.

### CDS prediction and functional annotation

The CDS and proteins encoded by all novel genes were predicted using ANGEL (https://github.com/PacificBiosciences/ANGEL) [91]. They were then functionally annotated by Blastx searches gainst the Nr, COG, Swiss-Prot, GO, RefSeq and KEGG databases with the cut-off e-value ≤1e-5 and highest score criteria.

### Identification of fusion transcripts in PacBio sequences

Fusion transcripts were identified using a Python script (fusion_finder.py) in the PBTRANSCRIPT-TOFU package (http://github.com/PacificBiosciences/cDNA_primer/) [40], with the following criteria: (a) mapping of a FLNC transcript to two or more annotated loci in the genome; (b) at least 10% of the transcript aligning with each mapped locus; (c) at least 99% combined alignment coverage; (d) at least 10 kb distance between each mapped locus; and (e) a certain amount of Illumina reads supporting the fusion regions [24].

### Differential expression gene analysis

Mapped reads of all genes were subjected to differential expression analysis using the edge R package [92]. Gene expression levels were calculated in terms of reads per kilobase of transcript sequence per million base pairs sequenced (FPKM) using stringTie [93]. The P.adjust function was used to adjust the generated *P*-value to control the false discovery rate. The threshold values for DEG significance were *P*-value ≤0.05 and absolute value log_2_ (group1/group2) ≥ 1. The DEGs were clustered based on FPKM by the gplots package (http://cran.r-project.org/web/packages/gplots/index.html), then functionally annotated by BlastX searches (e-value ≤1e-5) against the Nr, COG, Swiss-Prot, GO, RefSeq and KEGG databases. The online tool provided at https://sourceforge.net/p/enrichmentpipeline/wiki/Home/ was used for GO term and KEGG pathway enrichment analysis.

### Reverse transcription PCR (RT-PCR)

The RNA samples used for sequencing were also subjected to reverse transcription polymerase chain reaction (RT-PCR) analysis, with primers designed using primer-BLAST (https://www.ncbi.nlm.nih.gov/tools/primer-blast/). The PCR products were isolated and cloned into the PMD18-T Vector (Takara, Cat. #6011) for Sanger sequencing to validate PacBio isoforms.

### Real-time quantitative PCR (RT-qPCR)

cDNA was synthesized from 2 μg portions of total RNA using a PrimeScript RT reagent Kit with gDNA Eraser (TaKaRa, Cat. #RR047A), and SYBR green Supermix (Bio-Rad, Cat. #172-5274) was used to amplify cDNA. Real-time quantitative PCR was performed with a CFX96 real-time system, following the manufacturer’s instructions to verify the RNA-Seq analysis results. Sequences of primers used in real-time RT-PCR are listed in Additional file 6. BPH *actin* was used as an internal reference gene. At least three biological replicates were used in each experiment.

## Additional files

Additional file 1: **Figure S1.** Workflow of combined SMRT and RNA-seq analysis of BPH. (TIF 2045 kb)

Additional file 2: **Table S1.** Optimization of sequences’ structure. (XLSX 1879 kb)

Additional file 3: **Table S2.** Hits for unmapped and filtered PacBio reads in 15 species’ genomes with an E < 1e-5. (DOCX 15 kb)

Additional file 4: **Table S3.** Intron boundary sequences at 5′ and 3′ ends in the BPH genome. (XLSX 15 kb)

Additional file 5: **Table S4.** Alternative splicing events detected in female adults of BPH biotype 1. (XLSX 1015 kb)

Additional file 6: **Table S5.** All primers used in the study. (DOCX 22 kb)

Additional file 7: Long non-coding RNA identification. **Tables S6–S8**: All 2435 predicted long non-coding RNA sequences, 168 known long non-coding RNA sequences, and 2267 novel long non-coding RNA sequences. (XLSX 3777 kb)

Additional file 8: Detection and CDS of novel genes. **Tables S9.** and **S10**: All 1794 predicted novel gene sequences and 2664 CDS sequences for these predicted novel genes, respectively. (XLSX 3266 kb)

Additional file 9: Functional annotations of novel genes in six databases: Nr, Swiss-Prot, KEGG, GO, RefSeq and COG. (XLSX 382 kb)

Additional file 10: Detection of fusion genes. **Table S11.** All detected fusion genes’ sequences. **Table S12.** Positions of 915 fusion genes. (XLSX 1247 kb)

Additional file 11: Boundary sites of transcripts resulting from fusion of sequences of annotated genes at different locations. (XLSX 44 kb)

Additional file 12: **Figure S2.** Southern blotting analysis and resistance evaluation of T_2_ progeny of *Bph6-*transgenic rice plants. A, Southern blotting analysis of T_0_ progeny of *Bph6-* transgenic rice plants. The sample in the first lane indicated by the arrow is from a T0 line with a single-copy insertion, and the T2 homozygous genetic line used in this study originated from this line. B, Photograph of susceptible recipient rice Nipponbare and the T_2_ homozygous progeny of *Bph6-*transgenic rice after 5 days under BPH infestation. C, Amplification of *Bph6* cDNA sequences in constructed vector plasmid, *Bph6-*transgenic and Nipponbare rice plants. (TIF 5761 kb)

Additional file 13: **Figure S3.** Performance of BPH insects on susceptible and resistant rice plants. a-d, Survival rate, weight gain, honeydew excretion and weight gain ratio of BPH female adults fed on Nipponbare and *Bph6*-transgenic plants. Error bars represent SEM, *n* = 3 independent experiments, *P* values were derived from one-way ANOVA test. (TIF 617 kb)

Additional file 14: **Figure. S4.** Results of correlation analysis and Principal Component Analysis (PCA) of all RNA samples. (TIF 3096 kb)

Additional file 15: **Figure S5.** Volcano map of differentially expressed genes between N-BPH and NB6-BPH. Green dots represent significant differentially expressed genes; red dots represent non-significant differentially expressed genes. The significant difference criteria were fold change (FC) ≥ 2 and false discovery rate (FDR) < 0.01. (TIF 1124 kb)

Additional file 16: **Figure S6.** Hierarchical clustering analysis of 1893 DEGs based on the log ratio of FPKM. The color key represents FPKM-normalized log_2_ transformed counts by Z-score standardization. Red and green indicate up-regulated and down-regulated, respectively. Each column shows a sample and each row represents a gene. (TIF 178 kb)

Additional file 17: All 1893 DEGs between N-BPH and NB6-BPH. **Table S13.** DEGs up-regulated in NB6-BPH relative to N-BPH. **Table S14.** DEGs down-regulated in NB6-BPH relative to N-BPH. (XLSX 265 kb)

Additional file 18: GO enrichment of DEGs between N-BPH and NB6-BPH. **Table S15.** GO enrichment of DEGs up-regulated in NB6-BPH relative to N-BPH. **Table S16**. GO enrichment of DEGs down-regulated in NB6-BPH relative to N-BPH. (XLSX 27 kb)

Additional file 19: KEGG enrichment of DEGs between N-BPH and NB6-BPH. **Table S17.** KEGG enrichment of DEGs up-regulated in NB6-BPH relative to N-BPH. **Table S18.** KEGG enrichment of DEGs down-regulated in NB6-BPH relative to N-BPH. (XLSX 16 kb)

## Data Availability

The PacBio SMRT reads and Illumina RNA-seq reads supporting conclusions presented in this article are available in the NCBI Sequence Read Archive (SRA). The accession number for the SMRT reads is SRR8442724 under BioProject PRJNA514182. Accession numbers for the Illumina cDNA libraries obtained from the BPH biotype 1 fed on the susceptible and *Bph6*-transgenic rice plants for 48 h are SRR8437756, SRR8437757, SRR8437758, SRR8437759, SRR8437760, SRR8437761, SRR8437762 and SRR8437763 under BioProject PRJNA514182.

## Abbreviations

AltA: Alternative 3′ splice site
AltD: Alternative 5′ splice site
AltP: Alternative position
AMPK: AMP activated protein kinase
AS: Alternative splicing
ATG13: Autophagy-related protein 13
BNIP3: Bcl2/adenovirus EIB 19kD-interacting protein 3
BP: Biological process
BPH: Brown plant hopper
CarE: Carboxylesterases
CAT: Catalase
CC: Cell composition
CDS: Coding sequence
COG: Clusters of Orthologous Group
DEG: Differentially expressed gene
ES: Exon skipping
FC: Fold change
FDR: False discovery rate
FL: Full-length
FLNC: Full-length non-chimeric
GMAP: Genomic Mapping and Alignment Program
GO: Gene Ontology
GST: Glutathione S-transferase
ICE: Iterative Clustering for Error Correction
INRS: Insulin receptor substrate
IntronR: Intron retention
KEGG: Kyoto Encyclopedia of Genes and Genomics
lincRNAs: Long intergenic non-coding RNA
lncRNA: Long non-coding RNA
LoRDEC: Long Read DBG Error Correction
MF: Molecular function
MSR: Methionine sulfoxide reductase
NB6-BPH: BPH biotype 1 fed on *Bph6*-transgenic rice plants
N-BPH: BPH biotype 1 fed on susceptible rice Nipponbare plants
NGS: Next-generation sequencing
Nlbmm: *Nilaparvata lugens* brummer
Nr: Non-redundant
PCA: Principal Component Analysis
RNA-Seq: RNA sequencing
ROI: Read of insert
RT-PCR: Reverse transcription polymerase chain reaction
RT-qPCR: Real-time quantitative polymerase chain reaction
SMRT: Single-molecule, real-time
