# Supplementary material for: Combining next-generation sequencing and single-molecule sequencing to explore brown plant hopper responses to contrasting genotypes of japonica rice
Source: BMC Genomics. 2019 Aug 29;20:682. doi: 10.1186/s12864-019-6049-7 (PMC6716848; doi:10.1186/s12864-019-6049-7)
Supplement: Supplementary file 3 — Table S2. Hits for unmapped and filtered PacBio reads in 15 species’ genomes with an E < 1e-5. (DOCX 15 kb) [file 12864_2019_6049_MOESM3_ESM.docx]

| Species | Numbers of hit unmapped reads | Percentage of hit unmapped reads (%) | Numbers of hit filtered reads | Percentage of  hit filtered reads (%) |
| --- | --- | --- | --- | --- |
| *B. mori* | 232 | 12.56 | 19294 | 66.36 |
| 1. *plexippus* | 233 | 12.62 | 18964 | 65.23 |
| *A. gambiae* | 231 | 12.51 | 18580 | 63.91 |
| *A. aegypti* | 240 | 12.99 | 19472 | 66.98 |
| *D. melanogaster* | 225 | 12.18 | 18524 | 63.72 |
| *T. castaneum* | 261 | 14.13 | 19868 | 68.34 |
| *A. mellifera* | 238 | 12.89 | 4522 | 15.55 |
| *C. floridan* | 247 | 13.37 | 19876 | 68.37 |
| 1. *vitripennis* | 238 | 12.89 | 19533 | 67.19 |
| *P. humanus* | 231 | 12.51 | 19320 | 66.45 |
| *R. prolixus* | 82 | 4.44 | 11675 | 40.16 |
| *A. pisum* | 260 | 14.08 | 19583 | 67.36 |
| *YLS* | 22 | 1.19 | 4179 | 14.37 |
| *A. endosymbiont* | 261 | 14.13 | 2107 | 7.25 |
| *O. sativa* | 141 | 7.63 | 8933 | 30.73 |

**Table S2. Hits for unmapped and filtered PacBio reads hit in 15 species’ genomes**  **with E <1e-5.**
