# Supplementary material for: Combining next-generation sequencing and single-molecule sequencing to explore brown plant hopper responses to contrasting genotypes of japonica rice
Source: BMC Genomics. 2019 Aug 29;20:682. doi: 10.1186/s12864-019-6049-7 (PMC6716848; doi:10.1186/s12864-019-6049-7)
Supplement: Supplementary file 6 — Table S5. All primers used in the study. (DOCX 22 kb) [file 12864_2019_6049_MOESM6_ESM.docx]

**Table S5. All primers used in the study.**

| PCR primers of AS verification. | | |
| --- | --- | --- |
| Primer Name | Sequence (5'-3') | |
| PB.834-F | CAGAAGTCATGGAGGCGTTG | |
| PB.834-R | CTCTCATTCTCCTGGTCGGT | |
| PB.249-F | GGGATTGTGGAGGGCAAGAA | |
| PB.249-R | TTCGGTGTGCGTTATTCGGA | |
| PB.459-F | GTTCGCCTCAGAAATCGGTC | |
| PB.459-R | TCATACCGAACTCCAGCTCC | |
| PB.562-F | ATGGTGTGGAAGGTGTTTGC | |
| PB.562-R | AGTATTCCCACTCAACCCGG | |
| PB.688-F | CCAACCTGCCCAAGTACATG | |
| PB.688-R | AGATCTATTCAGGCATGTCACG | |
| PCR primers of fusion transcripts verification. | | |
| Primer Name | Sequence (5'-3') | |
| PBfusion.729-F | GATTCCTACCTTCGAGCGTCA | |
| PBfusion.729-R | TACTTTGAGAGCTGCGCGTC | |
| PBfusion.990-F | ATCCGCTAGTAGTGGTGTTTT | |
| PBfusion.990-R | GGATGGAGAACCTTGAAGGGA | |
| PBfusion.976-F | GCATGCGATTGATAGCTTGGA | |
| PBfusion.976-R | GCATTTACGGCGAAACGTGA | |
| PBfusion.2399-F | CACAGTCCAAACGCTCACAA | |
| PBfusion.2399-R | AGAAACCTGACGGCCTAACA | |
| PBfusion.1742-F | CTCCAAACCGTATCCGTATCA | |
| PBfusion.1742-R | CTCATCAGCGTTGAGTCAGA | |
| PBfusion.424-F | GGCTATCCCTGGCATCTCTC | |
| PBfusion.424-R | GGTACTTCCATCAAAATACAGAGCA | |
| PBfusion.2382-F | CGTACCGGTGCATCTCTAGT | |
| PBfusion.2382-R | GACATTCGCTCATACACTCCA | |
| PBfusion.2464-F | TGGATCTGGAAAGACAACGC | |
| PBfusion.2464-R | ACCATGTAGACCTGTGAGCC | |
| PBfusion.2405-F | ACTCGCCTCTTCACTGAACA | |
| PBfusion.2405-R | CCTGCATCTCCTCAAACACG | |
| RT-qPCR primers used to verify DEGs. | | |
| Primer Name | Sequence (5'-3') | Primer efficiency |
| *actin-F* | GACAGGATGCAGAAGGAAATCA | 86.40% |
| *actin-R* | GACTCGTCGTACTCCTGCTTTG |  |
| PB.6281-F | TGGCCTCAACCAACTGAACAA | 85.60% |
| PB.6281-R | TGCCTCTTGGCACTCAAACT |  |
| PB.96-F | GCACACAATACGGGACACAC | 92.10% |
| PB.96-R | GAAGCAGTTGTCACCGTTGC |  |
| PB.596-F | GCAGGAGTACGACAACGTCA | 98.00% |
| PB.596-R | TCACCCTTGAACAGTGTCCC |  |
| PB.2188-F | ACGAGGTCATCAAGCACGAG | 101.50% |
| PB.2188-R | CGATTACCATTGACGTACCGCT |  |
| PB.3946-F | CACTCCTCAACTACGCCCTG | 95.20% |
| PB.3946-R | TCTTCCCTTGTGAAGCTGCC |  |
| PB.6479-F | ATCTCAGGCGCAGTCAACAT | 95.50% |
| PB.6479-R | TTCCAAGGATGCGAGACACC |  |
| PB.6927-F | AGAAAGTTCGGACCGCCTAC | 100.50% |
| PB.6927-R | TCGAACCTTCTCTGGTATGACG |  |
| PB.7380-F | GCTTCGCGCACTTGTTAGAG | 97.40% |
| PB.7380-R | TGTAGGGTTGACAGAGCAGC |  |
| PB.7786-F | AGACACGCTAAAGTGGGCAA | 83.20% |
| PB.7786-R | GGCTCAACCACATCCCAACT |  |
| PB.9011-F | CTGCAACCACACCATGTTCG | 103.00% |
| PB.9011-R | AGTTGTTTCGCCTCGTGGAT |  |
| PB.1437-F | GGCTGGAATCGACCTTCCATA | 102.10% |
| PB.1437-R | ACTGCTGCAATCATCTTCGTT |  |
| PB.4152-F | TGGCTGATATCCGAACTGCC | 85.80% |
| PB.4152-R | GCTAAGGCTGGTTTCCATTAGC |  |
| gene1410-F | CTGTTTGCCTTGGTGAGTGC | 87.40% |
| gene1410-R | CATTGGTGTGTCCGGATGGA |  |
| gene3998-F | TAAGAGGCACGTCGTGACAG | 90.60% |
| gene3998-R | ACGCCTTTGAGTTGGTCAGT |  |
| PB.7873-F | GCGCTTGGATTGAGTATGCG | 81.20% |
| PB.7873-R | TGCTCATGTCTTCGGTAGGA |  |
| PB.7912-F | TAGAACTGCGCGAGGACATC | 94.30% |
| PB.7912-R | GTGGCCTCATTGGGCAAATC |  |
| PB.7996-F | CAGCAACTTCTTCGCACCAC | 82.60% |
| PB.7996-R | TCTTCTCAAACACTAGCAGACCA |  |
| gene8368-F | ATGACAACCACAGGGTAGCG | 103.40% |
| gene8368-R | GGTCTCGCCGTATGAGAGTG |  |
| PB.9218-F | CCGCTATTACTGTTGGTGCAG | 82.50% |
| PB.9218-R | TCCATCTCGTTTGAAATCTTCTCCT |  |
| gene17446-F | ATCACGCTGCTGTCTCTTGA | 96.40% |
| gene17446-R | AGTTGGAAAGCAGCCCACAT |  |
| gene862-F | GTCGCCACATACACCTGTGA | 85.50% |
| gene862-R | CGACTGCAGGTAGAGTCGTC |  |
| PB.912-F | CAAATGGCACAACGTAGCGG | 87.40% |
| PB.912-R | TGTCGAGAGAAGGTTGCACA |  |
| PB.3915-F | CCTCGCAGCTGATGGTTACT | 91.50% |
| PB.3915-R | TGAAGTGCTTATGATAGCAGATGG |  |
| gene5267-F | CGCATCTGTGTGTTCAAGCC | 102.90% |
| gene5267-R | CTCGTCCCTGATTTCGCCAT |  |
| PB.6424-F | TGTGGCGCTCAAGTCTGTAA | 96.70% |
| PB.6424-R | AAACTACAACGAGCGAGCAGA |  |
| PB.6848-F | ATGGGGTGCTTCTAGTTGGC | 101.40% |
| PB.6848-R | GAGGAACCACTGATCGCACA |  |
| gene10771-F | CACCGCTTACTGCATTGTCG | 91.10% |
| gene10771-R | CGAAGCTTTGAGGTTTCGGC |  |
| RT-qPCR primers of genes in foxO pathway. | | |
| Primer Name | Sequence (5'-3') | Primer efficiency |
| PB.6457-F | ACCTCACCTCTTCTGGTGAT | 92.90% |
| PB.6457-R | ACCCAATGCTCAATTGAAACG |  |
| PB.11835-F | GCTGCCTGGCTTCCAACTAA | 95.20% |
| PB.11835-R | CGGGTTGTAGCTGTAGGACG |  |
| PB.6002-F | ACTGTACTCACACAGCACCT | 100.10% |
| PB.6002-R | ACCGTTGATCCCAACCTTTCA |  |
| gene21312-F | GCGCCCAACTACTTCCCTAA | 91.40% |
| gene21312-R | GTGCTGAACTTGGTGTTGGC |  |
| PB.7786-F | AGACACGCTAAAGTGGGCAA | 83.20% |
| PB.7786-R | GGCTCAACCACATCCCAACT |  |
| PB.6927-F | AGAAAGTTCGGACCGCCTAC | 100.50% |
| PB.6927-R | TCGAACCTTCTCTGGTATGACG |  |
| PB.596-F | GCAGGAGTACGACAACGTCA | 98.00% |
| PB.596-R | TCACCCTTGAACAGTGTCCC |  |
| gene10945-F | CATTGCCCTACCTGTCCGTT | 93.40% |
| gene10945-R | TGAAGCAGATCACAGCGCAT |  |
| PB.6281-F | TGGCCTCAACCAACTGAACAA | 85.60% |
| PB.6281-R | TGCCTCTTGGCACTCAAACT |  |
| RT-qPCR primers of genes related to active oxygen elimination and detoxification. | | |
| Primer Name | Sequence (5'-3') | Primer efficiency |
| MSR-F | ACATTGTGCAAAGCTTGTCTACT | 100.10% |
| MSR-R | TCACATTATCCAATGCACAGAAGT |  |
| GST-like-F | CCGTTCAATGTGTCTCATC | 99.80% |
| GST-like-R | GGGTTTGTATCAAGTCATGG |  |
| GST-F | TCGGCAATTTTCACCCGAGA | 102.50% |
| GST-R | ATGTGTTCGCGCATGAATCG |  |
| CAT-F | ACTGTACTCACACAGCACCT | 100.10% |
| CAT-R | ACCGTTGATCCCAACCTTTCA |  |
| CAT-like-F | GCGCCCAACTACTTCCCTAA | 91.40% |
| CAT-like-R | GTGCTGAACTTGGTGTTGGC |  |
| ferrin-F | GCATGGCTTCAGTCTCCCTG | 92.90% |
| ferrin-R | GCGGCATCCTTCACTTCTTC |  |
| ferrin-like-F | TGAATGGTCATGGTAGATGTGT | 93.80% |
| ferrin-like-R | TGAATCACAGACAGTTGCAGT |  |
| CarE-F | ATGAGGGAGCTTTGGTTCTGG | 92.60% |
| CarE-R | AAATCTCCTTCGACCGGCAT |  |
| PCR primers of *Bph6* gene. | | |
| B6G-F | CGGGGTACCTGGAATCCGTTGGAAGTCTCC | |
| B6G-R | CGGGGTACCCCTGGCGGGACCTTTTGCGTT | |
| B6O-F | ATGCAGAGAAAGATTGCAGA | |
| B6O-R | CTAGGTACATGGTCCGATGC | |
