# Supplementary figures and images for: Combining next-generation sequencing and single-molecule sequencing to explore brown plant hopper responses to contrasting genotypes of japonica rice
Source: BMC Genomics. 2019 Aug 29;20:682. doi: 10.1186/s12864-019-6049-7 (PMC6716848; doi:10.1186/s12864-019-6049-7)

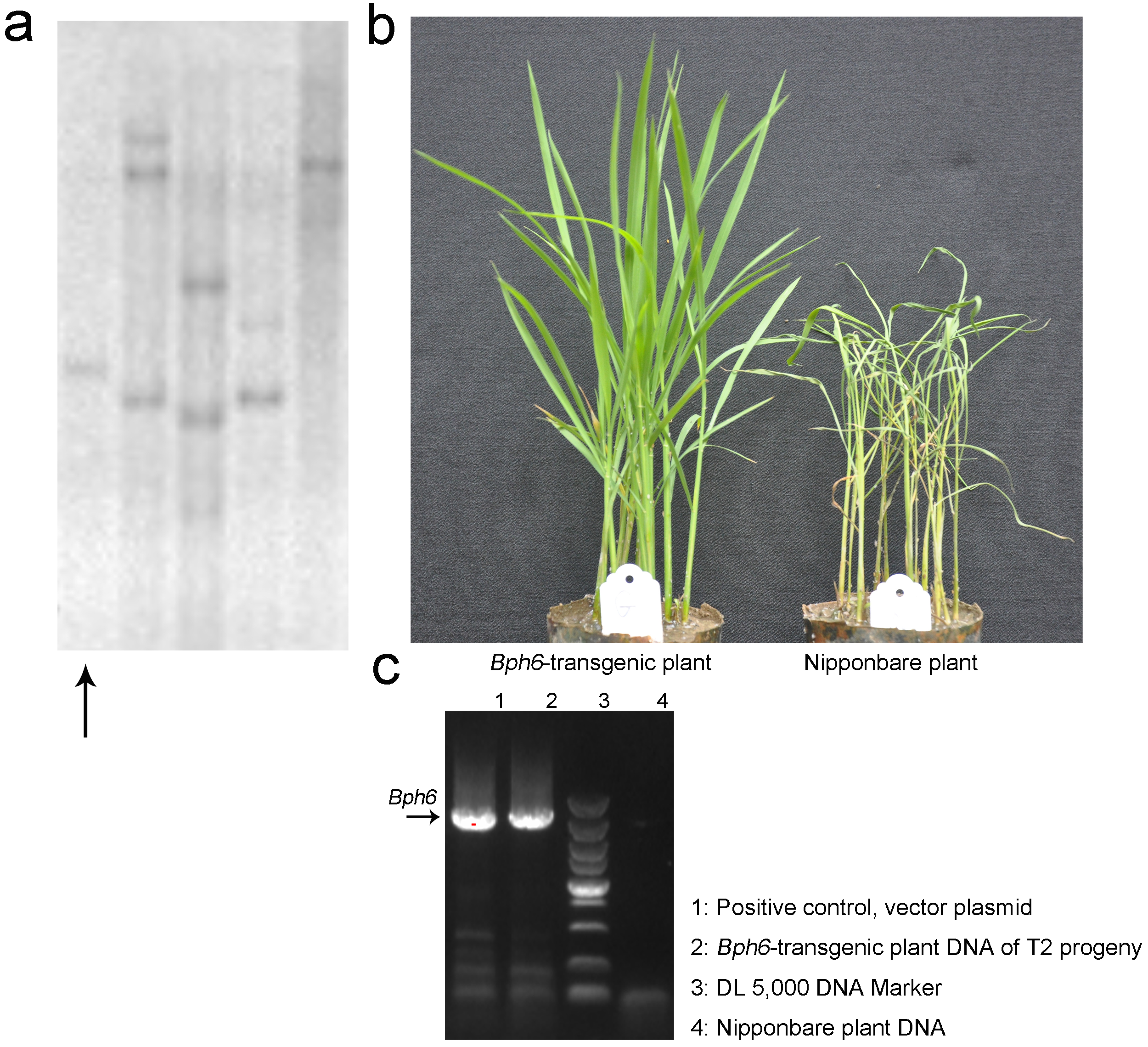

Supplement: Supplementary file 12 — Figure S2. Southern blotting analysis and resistance evaluation of T2 progeny of Bph6-transgenic rice plants. A, Southern blotting analysis of T0 progeny of Bph6- transgenic rice plants. The sample in the first lane indicated by the arrow is from a T0 line with a single-copy insertion, and the T2 homozygous genetic line used in this study originated from this line. B, Photograph of susceptible recipient rice Nipponbare and the T2 homozygous progeny of Bph6-transgenic rice after 5 days under BPH infestation. C, Amplification of Bph6 cDNA sequences in constructed vector plasmid, Bph6-transgenic and Nipponbare rice plants. (TIF 5761 kb) [file 12864_2019_6049_MOESM12_ESM.tif]

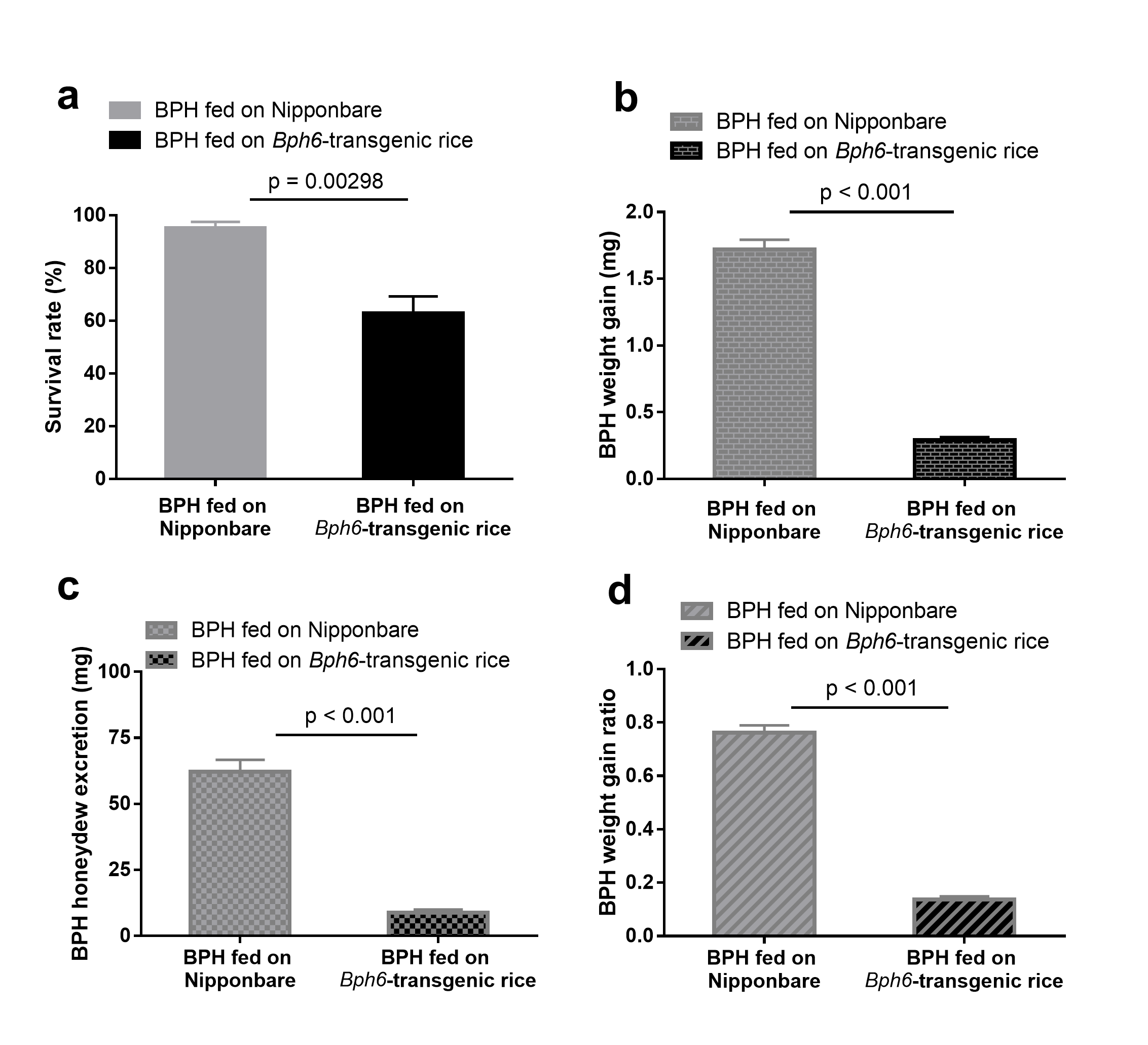

Supplement: Supplementary file 13 — Figure S3. Performance of BPH insects on susceptible and resistant rice plants. a-d, Survival rate, weight gain, honeydew excretion and weight gain ratio of BPH female adults fed on Nipponbare and Bph6-transgenic plants. Error bars represent SEM, n = 3 independent experiments, P values were derived from one-way ANOVA test. (TIF 617 kb) [file 12864_2019_6049_MOESM13_ESM.tif]

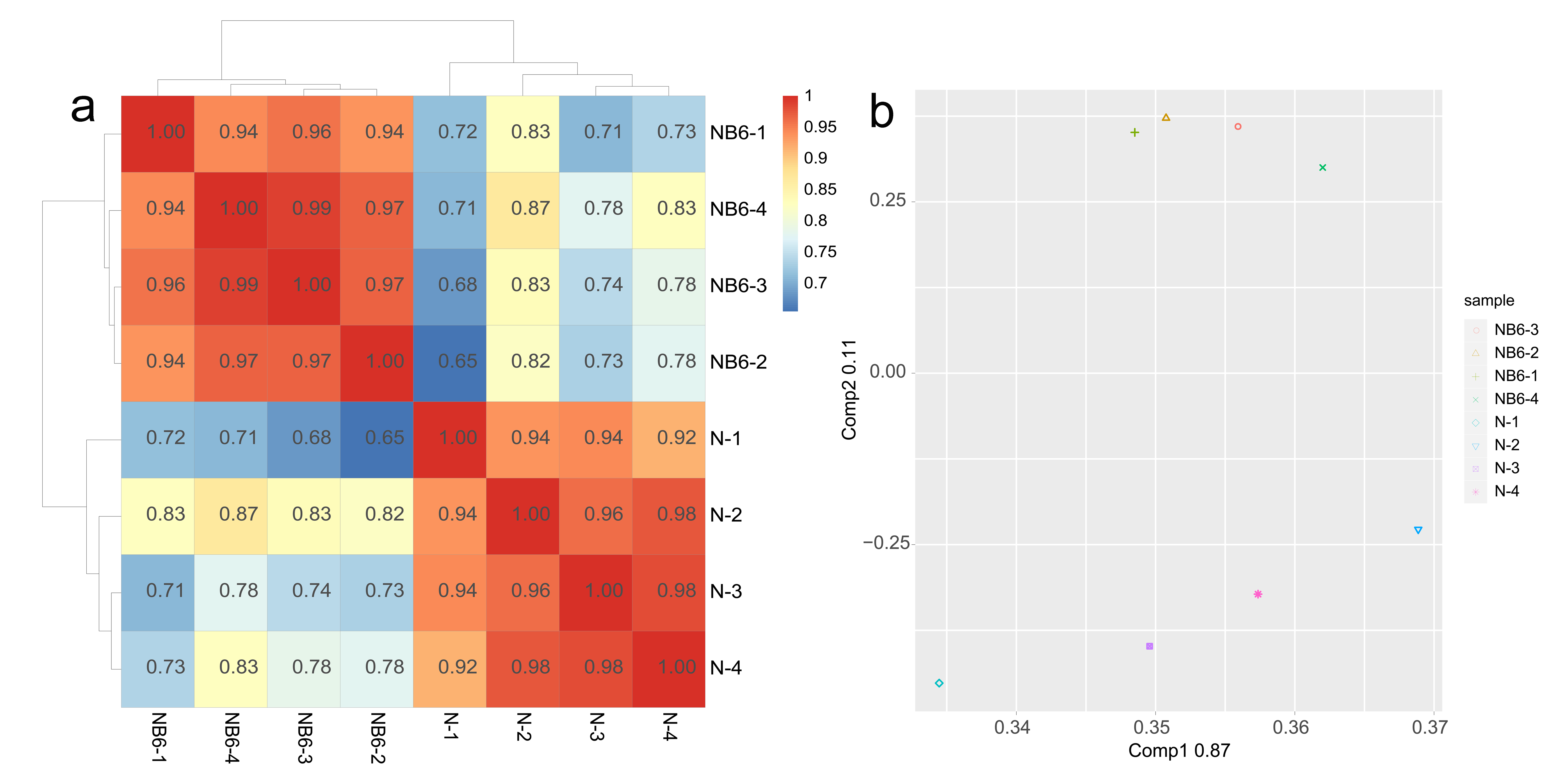

Supplement: Supplementary file 14 — Figure. S4. Results of correlation analysis and Principal Component Analysis (PCA) of all RNA samples. (TIF 3096 kb) [file 12864_2019_6049_MOESM14_ESM.tif]

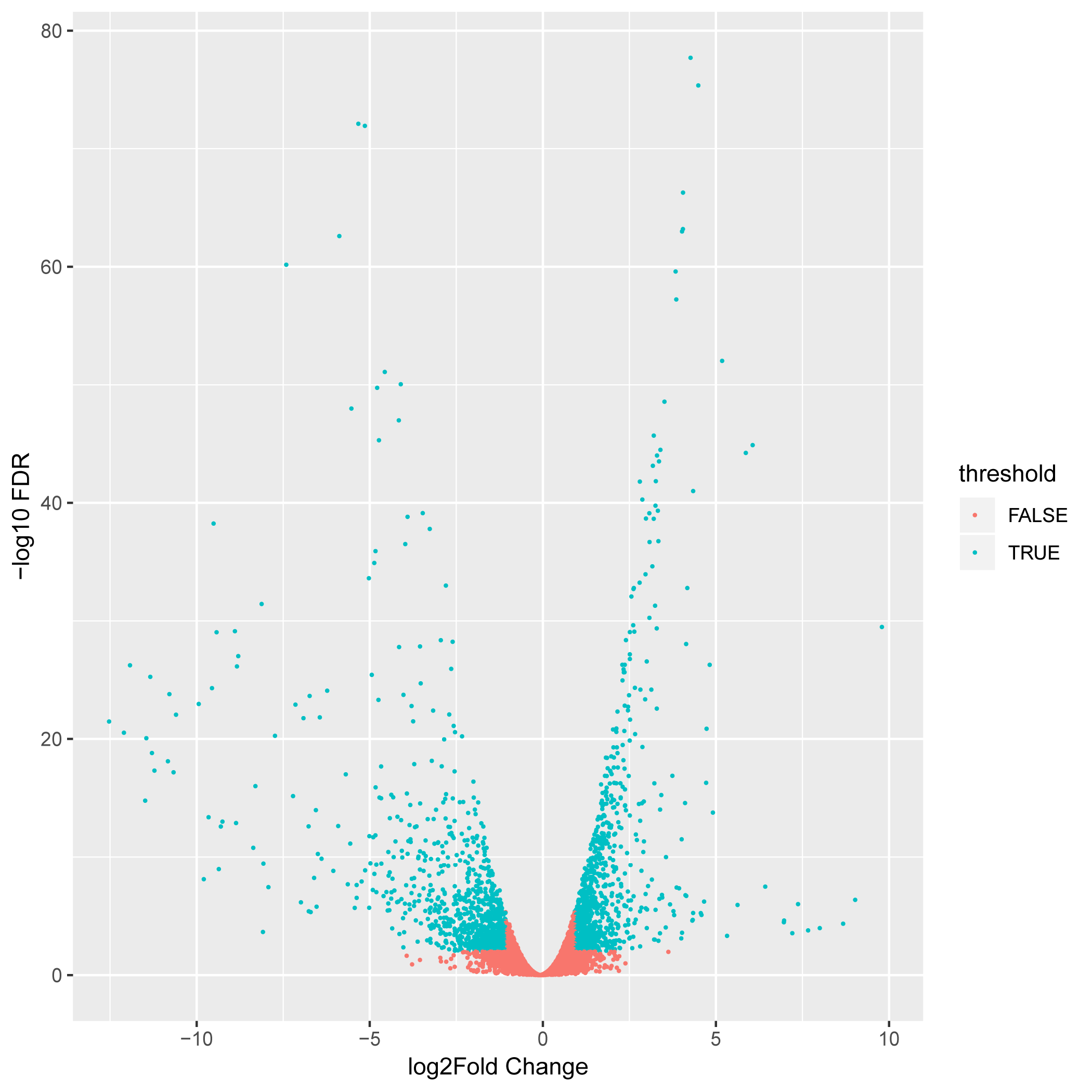

Supplement: Supplementary file 15 — Figure S5. Volcano map of differentially expressed genes between N-BPH and NB6-BPH. Green dots represent significant differentially expressed genes; red dots represent non-significant differentially expressed genes. The significant difference criteria were fold change (FC) ≥ 2 and false discovery rate (FDR) < 0.01. (TIF 1124 kb) [file 12864_2019_6049_MOESM15_ESM.tif]

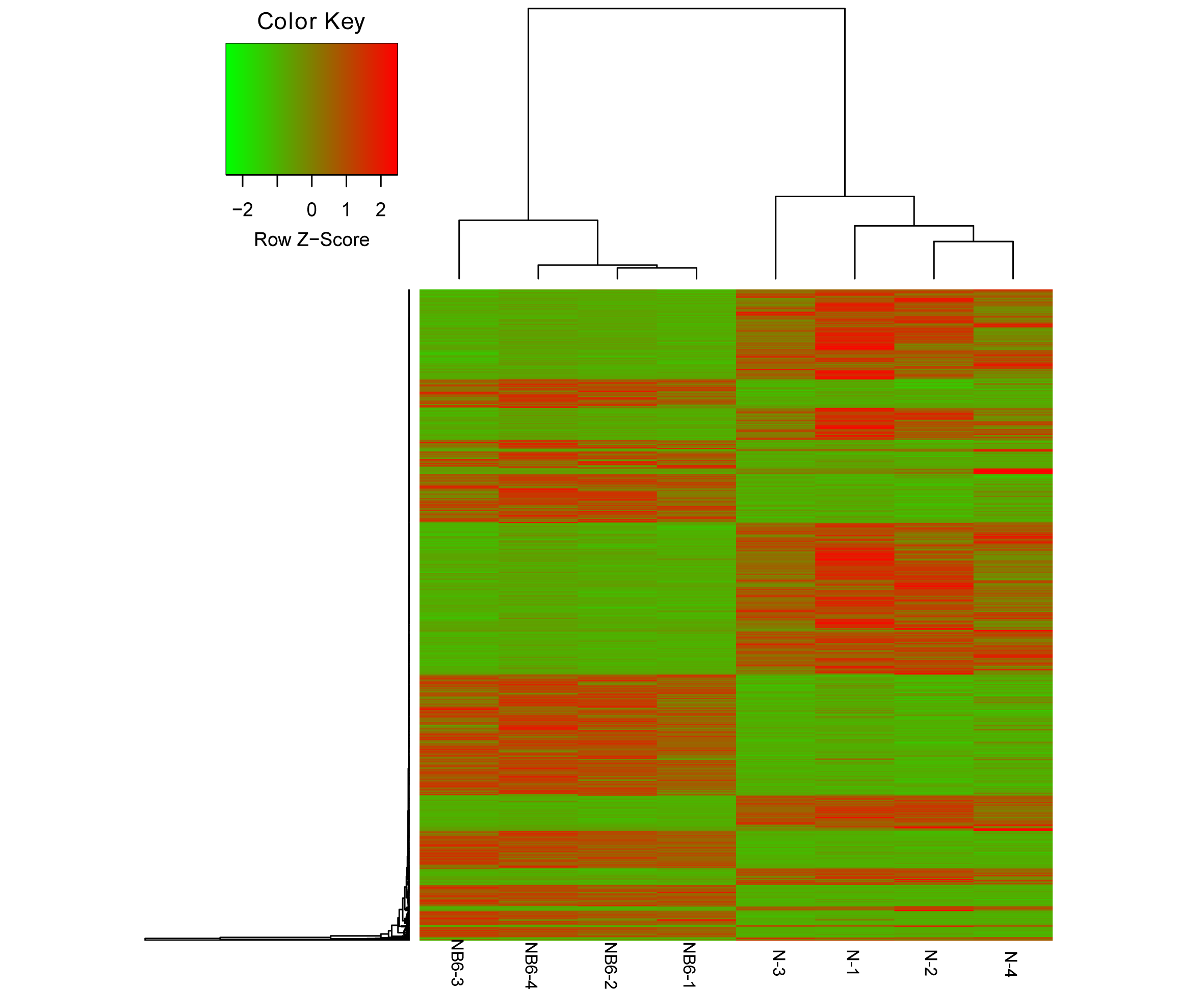

Supplement: Supplementary file 16 — Figure S6. Hierarchical clustering analysis of 1893 DEGs based on the log ratio of FPKM. The color key represents FPKM-normalized log2 transformed counts by Z-score standardization. Red and green indicate up-regulated and down-regulated, respectively. Each column shows a sample and each row represents a gene. (TIF 178 kb) [file 12864_2019_6049_MOESM16_ESM.tif]
